# Cilengitide sensitivity is predicted by overall integrin expression in breast cancer

**DOI:** 10.1186/s13058-024-01942-2

**Published:** 2024-12-20

**Authors:** Nomeda Girnius, Aylin Z. Henstridge, Benjamin Marks, Jeffrey K. Yu, G. Kenneth Gray, Chris Sander, Ioannis K. Zervantonakis, Augustin Luna

**Affiliations:** 1 Department of Cell Biology, Harvard Medical School, Boston, MA 02115 USA; 2 Department of Systems Biology, Harvard Medical School, Boston, MA 02115 USA; 3 Department of Bioengineering, Swanson School of Engineering, University of Pittsburgh, Pittsburgh, PA 15213 USA; 4 Computational Biology Branch, National Library of Medicine and Developmental Therapeutics Branch, National Cancer Institute, Bethesda, MD 20892 USA; 5 Laboratory of Systems Pharmacology, Harvard Medical School, Boston, USA

**Keywords:** Breast cancer, Triple negative breast cancer, Integrins, Proteomics, Extracellular matrix, Drug screening

## Abstract

**Background:**

Treatment options for triple-negative breast cancer (TNBC) are limited and patients face a poor prognosis. Here, we sought to identify drugs that target TNBC vulnerabilities and understand the biology underlying these responses. We analyzed the Broad Institute DepMap to identify recurrent TNBC vulnerabilities and performed a 45-compound screen on vulnerability-related pathways on a set of up to 8 TNBC cell lines. We identified a subset of cell lines with an ITGAV vulnerability and a differential sensitivity to cilengitide, an integrin inhibitor targeting ITGAV:ITGB3 and ITGAV:ITGB5. Next, we sought to understand cilengitide resistance and response biomarkers. Clinical trials targeting integrins continue enrolling patients, necessitating an understanding of how these drugs affect tumors.

**Methods:**

We combined in vitro assays with computational approaches to systematically explore the differential sensitivity to cilengitide and resistance mechanisms. We tested an additional pan-ITGAV inhibitor (GLPG0187) to determine how generalizable our findings on cilengitide sensitivity might be to integrin inhibition. ITGB4, ITGA3, and ITGA6 knockdown experiments assessed the importance of integrin monomers in cell attachment during cilengitide treatment. Additionally, we explored the role of extracellular matrix (ECM) proteins in cilengitide response by performing cell replating experiments and by culturing on collagen, fibronectin, or laminin coated plates.

**Results:**

We discovered that cell-derived ECM modulates cilengitide sensitivity and exogenous fibronectin addition conferred resistance to all sensitive TNBC cell lines, though fibronectin expression did not correlate with sensitivity. Instead, elevated overall integrin protein levels, not specific integrins, in TNBC cells positively correlated with resistance. This suggested that high pan-integrin expression promotes cilengitide resistance. Thus, we tested cilengitide in six luminal breast cancer cell lines (which have low integrin levels); all were sensitive. Also, pan-ITGAV inhibitor, GLPG0187, showed the same sensitivity profile across our TNBC cell lines, suggesting our findings apply to other integrin inhibitors.

**Conclusions:**

Integrin inhibitors are appealing candidates to pursue as anti-cancer drugs because they are generally well-tolerated, but their efficacy is mixed, possibly due to the absence of predictive markers. Cilengitide induces death in breast cancer cells with low integrin abundance, where complementary ECM promotes survival. Thus, integrin inhibition in breast cancer warrants further study.

**Supplementary Information:**

The online version contains supplementary material available at 10.1186/s13058-024-01942-2.

## Background

TNBC accounts for 10–15% of breast cancer patients, and disproportionately affects African-American and Hispanic women [1, 2]. TNBC is an especially malignant subtype characterized by high invasiveness and metastatic potential, poor prognosis, and few treatment options due to an absence of well-defined targetable alterations [3–6]. Only a small portion of patients with TNBC (~ 20%) respond well to standard therapy (i.e., surgery, radiation, and chemotherapy) [7]. Using genetic screens to identify cancer dependencies that can be targeted with existing drugs to test rational drug candidates could be an approach to meet this critical need for TNBC [8]. Project Achilles (Broad DepMap) is a CRISPR screen from the Broad Institute [8], which at the start of this study contained dependency scores for 26 breast cancer cell lines, including 13 TNBC lines. In this work, we used the DepMap dataset to find unique dependencies in TNBC cell lines, then identified 45 drugs to target those dependencies and related pathways of interest which were tested for efficacy in up to 8 TNBC cell lines representing basal A and basal B subtypes. Of the drugs we screened, we found the integrin inhibitor cilengitide induced strong differential effects across cell lines.

Targeting integrins is an appealing strategy in breast cancer. The integrin pair ITGAV:ITGB3 is expressed in breast cancer and can mediate metastasis to the bone [9]. Perhaps relatedly, high expression of ITGAV is associated with a poor prognosis for patients with metastatic breast cancer [10]. Several integrin inhibitors have been developed including cilengitide, a cyclic RGD motif-containing pentapeptide designed to inhibit the interaction of ECM RGD motifs with the binding pocket of ITGAV:ITGB3 (i.e., αvβ3) and ITGAV:ITGB5 (i.e., αvβ5) integrin pairs that bind primarily to fibronectin and fibrinogen [11]. Despite its intended use as an inhibitor of angiogenesis [12, 13], cilengitide has been reported to have anti-tumor cell activities outside of its effects on tumor vasculature. Mice treated with cilengitide following breast cell engraftment had decreased metastatic area in the lung [10] compared to untreated animals. Treatment of MDAMB231 breast cancer cells with cilengitide has also been shown to reverse their mesenchymal phenotype, increasing their sensitivity to paclitaxel [14]. Furthermore, the ITGAV:ITGB3 integrin pair plays a role in cell survival following radiation therapy in both prostate cancer [15] and breast cancer [16, 17]. These together with many other preclinical studies suggested that targeting integrins represents an actionable therapeutic vulnerability.

While cilengitide and other integrin inhibitors have been well-tolerated in clinical trials [18–21], their overall performance treating cancer has been mixed. In a phase I trial for solid tumors, one metastatic TNBC patient showed a partial response when cilengitide was combined with paclitaxel [18]. In glioblastoma (GBM), data from phase I and II trials of cilengitide showed promising results [22], but the phase III CENTRIC trial (NCT00689221) failed to meet overall survival endpoints [23]. Still, more recent retrospective analysis of the CENTRIC study suggests cilengitide treatment may delay progressive disease and prolong patient survival [24]. Additionally, the small molecule GLPG0187 was well-tolerated in a phase I study, but failed to show efficacy as a single therapy [21]. The mixed performance of integrin inhibitors is possibly due to suboptimal dosage and an absence of biomarkers to target appropriate patient populations [25, 26]. In addition to the overall complexity of cell adhesion [27, 28], progress of anti-integrin therapeutics in cancer is hampered by (1) variable integrin expression in tumors, (2) redundancy in integrin function, and (3) changes in the roles of integrins at different disease stages [29]. Nevertheless, clinical studies using integrin inhibitors continue being developed and are enrolling patients (e.g., NCT05085548, NCT06603844). An important step forward in the field of integrin inhibition would be the discovery of biomarkers to identify patients most likely to benefit from this treatment approach [11, 23].

In our drug screen across a panel of TNBC cell lines, cilengitide induced cytotoxicity in half of the cell lines, motivating a systematic approach to find characteristics of sensitive cells that may serve as biomarkers for therapeutic response prediction. We have previously performed similar analyses in the context of high-grade serous ovarian cancer [30]. Because of the relatively frequent lack of correlation between mRNA and protein, we focused on proteomics data in this study. Combining computational and experimental approaches with drug response profiling we explored protein expression differences and their influence on cilengitide sensitivity in TNBC. We found that cellular adhesion pathways were upregulated in resistant cell lines. Several integrins, in particular ITGA6 and ITGB4, which are not targeted by cilengitide, were more abundant in resistant compared to sensitive cell lines. Meanwhile, the protein levels of extracellular matrix (ECM) proteins which can be bound by integrins were not markedly different across the two response groups. Interestingly, we further demonstrate that overall low levels of integrins predicts sensitivity to cilengitide across breast cancer subtypes, as luminal breast cancers, which have low integrin levels, are also sensitive to this compound. By systematically cataloging differences in patterns of breast cancer integrin expression and dependency, and putting these findings in the context of the extracellular matrix proteins, our analyses provide new insight into mechanisms of cilengitide resistance.

## Results

### Identification and targeting of TNBC vulnerabilities reveal differential drug sensitivity

**Fig. 1:**
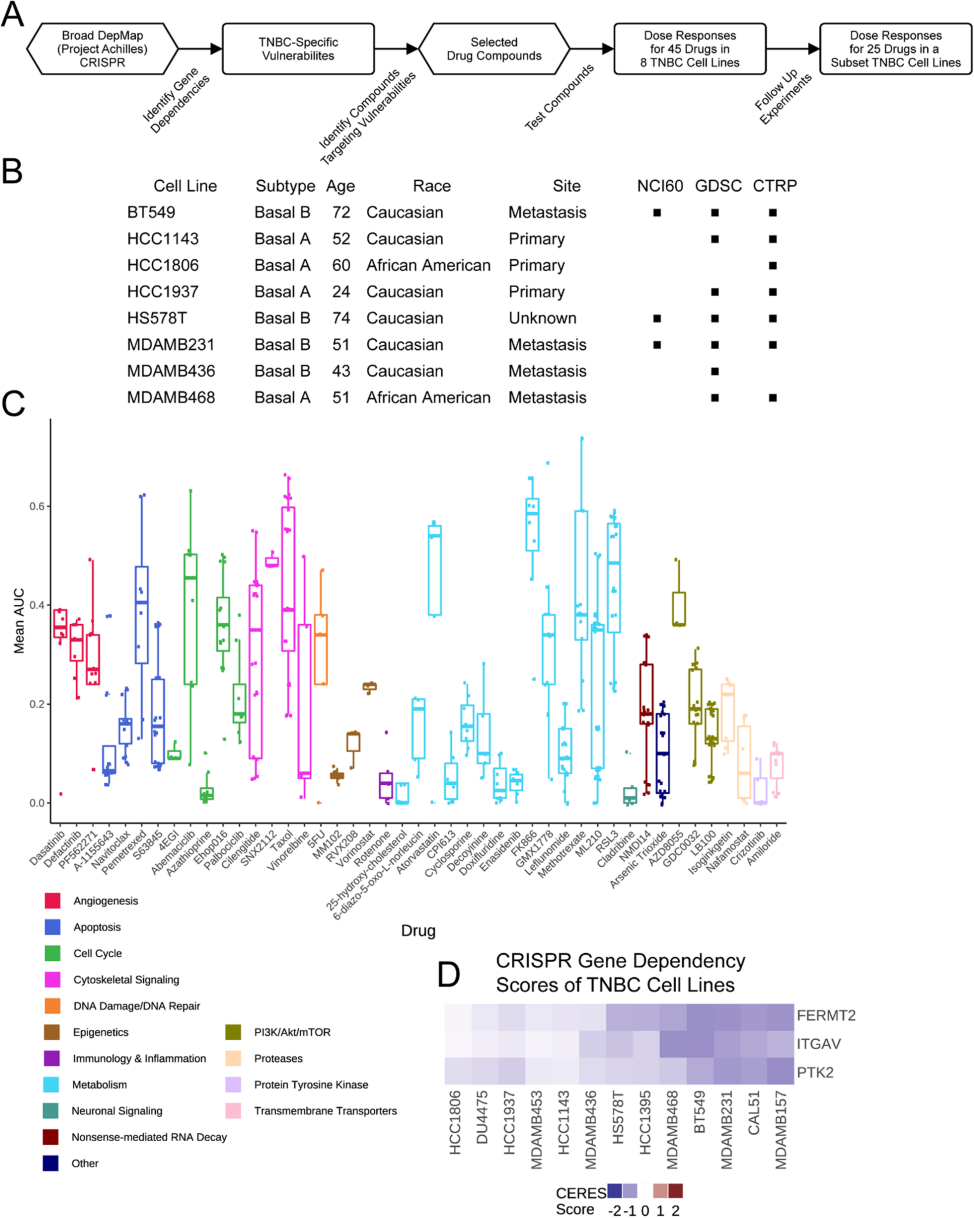
A drug treatment dataset designed using the DepMap applied to TNBC cell lines. **A**. Workflow of drug panel design and application. **B**. Description of cell lines used to generate the drug treatment dataset, including their subtype, patient’s age and race, the site of collection, and the presence (black square) of the cell line in other drug screening datasets. **C**. AUC values of all screened drugs are shown as a boxplot which displays a five-number summary, including the minimum, first quartile, median, third quartile, and maximum AUC, color-coded by mechanism of action. **D**. Cell line gene dependency values from the DepMap for FERMT2, PTK2, and ITGAV are shown. Dependency scores of -0.7 or lower are considered dependent

We queried the gene dependency data from the Broad DepMap dataset for genes with a variable range of dependency (i.e., vulnerabilities in at least two, but not all, breast cancer cell lines), enriched in TNBC cell lines compared to other breast tumor subtypes, with CERES dependency scores of -0.7 or lower. From these dependencies, we constructed a 45-drug panel composed of compounds that would inhibit either the gene product or related cellular process of those dependencies (Fig. 1A and Supp. Table 1 & 2). A diverse panel of eight cell lines representing basal A and basal B subtypes, derived from primary or metastatic sites, and including two African American-derived lines was assembled for our drug screening (Fig. 1B). Inclusion of these cell lines in other drug treatment datasets was noted (Fig. 1B, black square). Nine-point dose response curves were fitted and the area under the curve (AUC) was calculated (Supp. Table 3) for each cell line-drug pair such that higher AUC values correspond to higher drug sensitivity (i.e., 1-Viability area; see Methods). Notably, our dataset adds 18 drugs not previously tested in TNBC as part of large, public screening efforts (comparison via CellMinerCDB, which aggregates pharmacogenomics datasets from multiple institutes) complementing the existing body of work in this area [31].

The tested compounds produced a range of responses across the cell lines (Fig. 1C, see Methods). Several drugs had a consistent effect across all cell lines (e.g., dasatinib, A1155643, MM102, vorinostat, etc.), meaning the AUC values were tightly grouped, not exhibiting a differential response that would be amenable for biomarker identification. The unanticipated lack of variable responses to these compounds may relate to the time scale and specificity of inhibition using CRISPR instead of a drug. Other drugs, however, showed a wide range of AUC values (e.g., pemetrexed, abemaciclib, cilengitide, vinorelbine, methotrexate, and ML210) demonstrating that gene dependencies can help identify targetable pathways and processes that represent cell vulnerabilities. This group of compounds is more amenable to statistical analysis to identify biomarkers that might stratify patients by benefit. We performed validation studies on the 25 drugs that showed the greatest heterogeneity in our primary screen and found that cilengitide, an inhibitor of the ITGAV:ITGB3 integrin heterodimer, had a robust, reproducible phenotype across experiments (Spearman correlation between replicates = 0.67).

Cilengitide was included in our drug screen to target observed dependencies (based on the CERES metric developed by the Broad Institute [8]) on three proteins involved in cell adhesion: FERMT2, ITGAV, and PTK2. Seven out of 13 TNBC cell lines were dependent on FERMT2, which is also known as kindlin-2, an integrin co-activator that mediates signaling between integrins and the focal-adhesion kinase pathway (upstream of PI3K/AKT/mTOR) [32–35]. Of the FERMT2-dependent lines, six were also dependent on ITGAV and four of those were dependent on PTK2, the gene that encodes the focal adhesion kinase (Fig. 1D), suggesting that some TNBC cell lines are especially dependent on the focal adhesion complex involved in cell adhesion. Given our observations of the differential dependencies in DepMap and drug responses in our dataset, we chose to use a systematic approach to understand TNBC cell response to ITGAV inhibition by cilengitide.

### TNBC cell lines exhibit differential sensitivity to the integrin inhibitor cilengitide

**Fig. 2:**
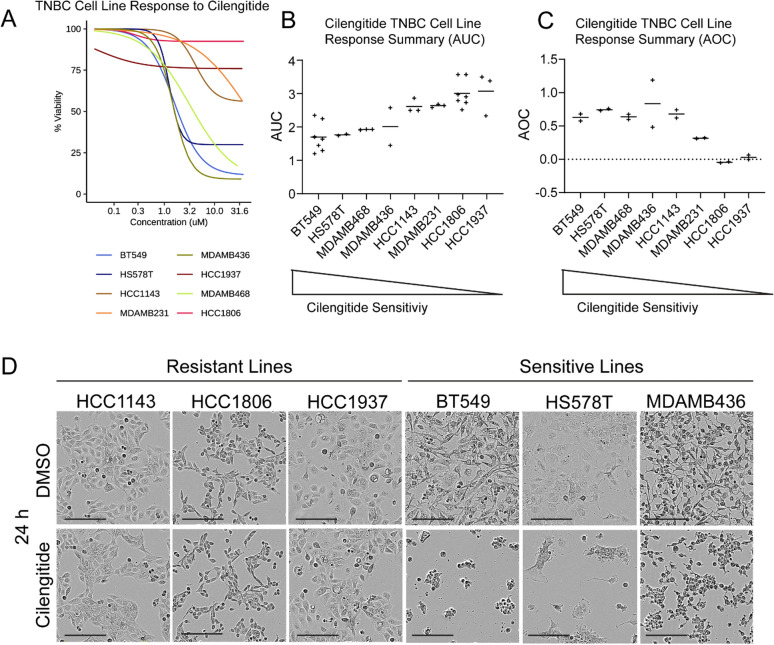
Some TNBC cell lines are dependent on adhesion proteins and respond to the integrin inhibitor, cilengitide. **A & B**. Dose response curves were fitted based on 9 doses of cilengitide for 8 TNBC cell lines. Representative results from 1 of 4 experiments are shown (**A**). The AUC values from replicate experiments together with the mean (horizontal bar) were plotted in order from most to least sensitive (**B**). **C**. GR metrics were calculated for the 8 TNBC cell lines treated with cilengitide, and AOC values from replicate experiments were plotted together with the mean (horizontal bar). Higher AOC values indicate increased sensitivity. **D**. Bright field images showing the morphology of cells at 24 h post-treatment with DMSO or cilengitide (5 µM) are shown (scale bar = 200 μm)

We grouped cell lines that had an IC50 value below 5 μM as sensitive (BT549, HS578T, MDAMB436, MDAMB468), while cells with an IC50 value over 5 μM were classified as resistant (HCC1806, HCC1937, HCC1143, MDAMB231), resulting in two equal groups of cell lines (Fig. 2A). Using conventional methods of measuring cell line drug sensitivity, area-under-the-curve (AUC) values were computed (Fig. 2B). Since growth rate is known to confound comparative drug sensitivity studies across cell lines [36], Growth Rate inhibition values (GR) were calculated and compared to the AUC. We found that both the area-over-the-curve (AOC; Fig. 2C) and AUC demonstrated striking resistance in HCC1937 and HCC1806 lines and placed BT549 and HS578T as among the most sensitive lines. An examination of cell morphology across the cell lines after 24 h of treatment revealed that BT549, HS578T, and MDAMB436 detached in the presence of cilengitide, whereas HCC1143, HCC1806, and HCC1937 all remained attached (Fig. 2D). Thus, while the IC50, AUC, and AOC metrics for line HCC1143 presented a mixed phenotype, the line’s continued attachment (Fig. 2D) and proliferation (Supp. Figure 1A) in the presence of cilengitide, in contrast to other sensitive lines (Supp. Figure 1B), led to its classification as a resistant cell line. To assess how consistent the sensitive and resistant cell line classifications would be with ITGAV inhibition, we examined TNBC cell proliferation in the presence of the small molecule, pan-ITGAV inhibitor GLPG0187, which was also well-tolerated in phase I trials [21]. We found that the cells’ designation of sensitive and resistant to cilengitide was consistent with response to GLPG0187 (Supp. Figure 1C and D).

To understand the nature of the cell death induced by cilengitide in the sensitive lines, we cultured them in the presence of a caspase 3/7-activatable fluorescent dye. If cells engaged the caspase-mediated apoptotic pathway following detachment, it would indicate that they are undergoing anoikis [37]. Indeed, the sensitive cell lines all underwent detachment and showed caspase 3 activation by twelve hours or earlier, suggesting that the sensitive cells undergo apoptotic cell death in response to cilengitide-induced suspension (Supp. Figure 1E). Indeed, we noticed that in lines BT549 and HS578T, the detachment and fluorescence occurred even more rapidly, making it difficult to establish the order of events.

The dependencies on PTK2, FERMT2, and ITGAV observed in the DepMap had suggested that certain TNBC cell lines would be sensitive to inhibition of integrin-mediated adhesion. To assess whether the CRISPR vulnerabilities followed the same pattern of cilengitide sensitivity observed across the cell lines, we checked the sensitive and resistant cell line CRISPR dependencies from the DepMap. Of the three genes, ITGAV had the most striking difference in the CERES scores between the resistant and sensitive lines tested, though it did not reach significance (Supp Fig. 1F). Just as PTK2 dependency did not follow cilengitide sensitivity, FAK inhibition also failed to correlate with this characteristic. Two different FAK inhibitors (PF-562271 and GSK2256098C) in the Genomics of Drug Sensitivity in Cancer (GDSC) cell line drug screening database showed no relationship with cilengitide sensitivity (-0.39 and -0.18, respectively and neither was statistically significant using the available samples, Supp. Table 4). Unlike PTK2 or FERMT2, ITGAV is a direct target of cilengitide [12, 13]. Thus, the observed dependency on ITGAV largely translated to sensitivity to ITGAV inhibition in TNBC separate from the downstream effectors PTK2 and FERMT2.

### Proteins involved in cell adhesion are differentially abundant between cilengitide sensitive and resistant lines

Currently, there exist contradictory reports regarding the role of target integrin expression and the response to cilengitide. In a retrospective study using patient tissues from the CORE and CENTRIC trials, which tested the efficacy of cilengitide in glioblastoma, the expression of target integrins was associated with clinical response to cilengitide: improved progression-free survival was reported in glioblastoma patients whose tumors expressed ITGAV:ITGB3 [38]. However, a separate in vitro study showed that breast cancer cell lines with higher expression of ITGB3 were resistant and could be sensitized by knockdown of ITGB3 to lower levels [16]. These data raised the possibility that a cilengitide-sensitive cell will express the target integrins at levels allowing effective inhibition.

**Fig. 3:**
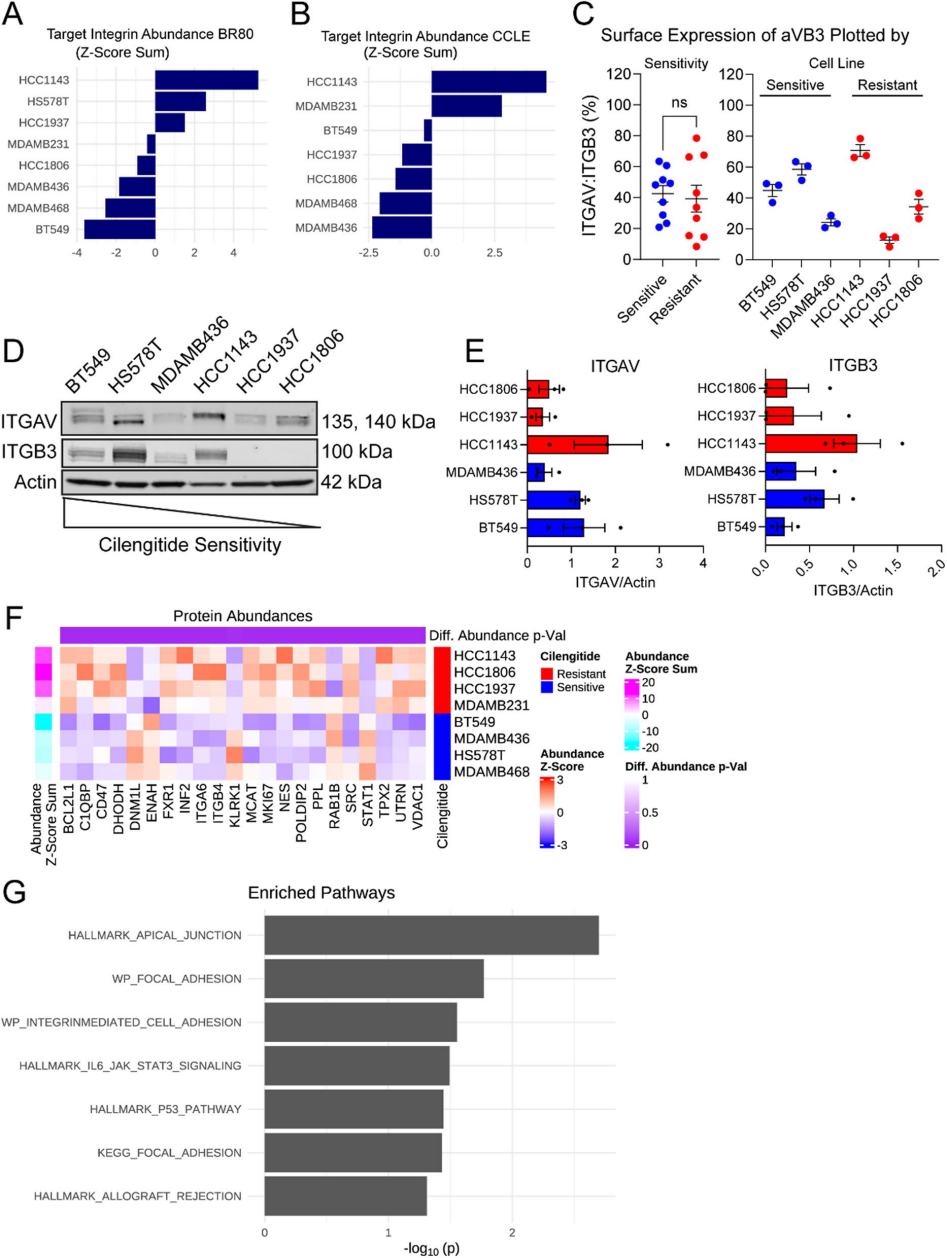
Elevated cell adhesion proteins are a distinguishing characteristic of cilengitide resistant and sensitive lines. **A** & **B**. Bar graphs of summed z-scores ranked from the highest to lowest sum for the cilengitide target integrins ITGAV and ITGB5 from the BR80 (**A**) and ITGAV, ITGB3, and ITGB5 from the CCLE (**B**) proteomics datasets are shown. **C**. Surface-expressed ITGAV:ITGB3 was assessed using flow cytometry and percentages of positive cells were quantified for each cell line, then plotted by sensitivity to cilengitide (left) or by cell line (right). No significant (ns) difference was found (t-test) between the sensitive and resistant lines. The mean with S.E.M. is shown. **D**. Protein levels of cilengitide targets ITGAV and ITGB3 were probed by immunoblot, with beta-actin (same blot as in Fig. 4F) serving as a loading control. Cell lines were loaded from left to right by their cilengitide sensitivity (most to least resistant). **E**. Three independent cell protein lysates from each cell line were quantified for ITGAV and ITGB3 and normalized to beta-actin. Resistant lines are colored in red, while sensitive lines are blue. Mean with S.E.M. is shown. **F**. TNBC-associated proteins (DisGeNET) were assessed for differential protein abundance (p < 0.05, unadjusted Wilcoxon signed rank) in the BR80 dataset between resistant (red) and sensitive (blue) lines and the abundance of the 7 proteins meeting the cutoff is shown as a heatmap with higher abundance in red and lower in blue. **G**. Differential protein abundance of the resistant and sensitive cell lines was determined across the entire BR80 proteomics dataset and used to perform gene set enrichment analysis (see Methods). The -log of the p-value is plotted for pathways that reached significance (p < 0.01)

Thus, we examined cilengitide target expression in two proteomics datasets: the BR80 [39] and CCLE [40], which together covered all of our cell lines and more integrins than either dataset alone. We calculated z-scores for the abundances provided in each dataset to facilitate comparison. The z-scores of the target integrins were added together to calculate an overall summary score and ranked from highest to lowest (Fig. 3A). In the BR80 dataset, the resistant line HCC1143 had the highest abundance of ITGAV and ITGB5, followed by the sensitive line HS578T. Furthermore, the cell lines with intermediate expression were all from the resistant group. In the CCLE dataset, which lacked the sensitive line HS578T, the resistant lines HCC1143 and MDAMB231 ranked highest for the z-score sum of ITGAV, ITGB3, and ITGB5 (Fig. 3B). Meanwhile, HCC1806 and HCC1937, which had the highest IC50 and GR_Max_ response values, had lower expression (i.e., lower overall summary score) of the cilengitide targets than the sensitive line BT549, but higher expression than two other sensitive lines. To further validate these findings and to assay ITGB3 expression (no data in BR80) in HS578T cells (no data in CCLE), we performed flow cytometry and immunoblotting experiments to check integrin abundance. Using an antibody against the extracellular portion of the ITGAV:ITGB3 dimer, we could show that there was no significant difference in surface expression of this dimer between sensitive and resistant lines, and that HCC1143 and HS578T had the highest levels of this dimer (Fig. 3C). Immunofluorescent ITGAV:ITGB3 dimer staining confirmed similar punctate staining across cell lines (Supp. Fig. 2). Blotting experiments further supported the observation that HCC1143 was the highest ITGAV-expressing line (Fig. 3D and E), in agreement with the BR80 and CCLE datasets. Meanwhile, the resistant line HCC1143 and the sensitive line HS578T were the top expressors of ITGB3. Taken together, we found no relationship between cilengitide response and ITGAV or ITGB3 abundance, suggesting that at the protein level neither ITGAV nor ITGB3 could predict TNBC response to ITGAV inhibition.

Next, we evaluated more broadly protein expression patterns that may account for cilengitide resistance. We identified 22 proteins associated with TNBC (taken from DisGeNET, one of the largest databases of genes associated with human disease) that were present in the BR80 dataset and differentially abundant (unadjusted p < 0.05) between the sensitive and resistant lines (Fig. 3F). Among the identified proteins were ITGA6 and ITGB4; while neither is reported to be a target of cilengitide, it raised the possibility that integrins other than ITGAV and ITGB3 may modulate cilengitide sensitivity. Another of the TNBC-specific proteins identified, SRC, lent support to the hypothesis that upregulation of adhesion complex members may decrease cell response to cilengitide. To explore this possibility further, we performed GSEA using the MSigDB Hallmark signatures together with KEGG and WikiPathway adhesion gene set signatures on all differentially abundant proteins in the BR80 dataset across the two groups of cell lines. This analysis showed that proteins in the WikiPathways Focal Adhesion and Integrin-mediated Adhesion as well as the KEGG Focal Adhesion were enriched in the resistant cell lines (Fig. 3G). Furthermore, the leading-edge genes (i.e., genes contributing the most to the enrichment) in our analysis (Supp. Table 5) consisted of ECM proteins (e.g., FBN1, LAMB3, and COL4A2), integrins (e.g., ITGA2, ITGA3, ITGA6, ITGB1, and ITGB4), and proteins involved in the focal adhesion complex and its downstream signaling (e.g., ZYX, SRC, RAC1, ROCK1). These results indicated that differences in cell adhesion between sensitive and resistant cells mediate the response to ITGAV inhibition.

### Integrin and ECM protein abundance in TNBC cell lines

**Fig. 4:**
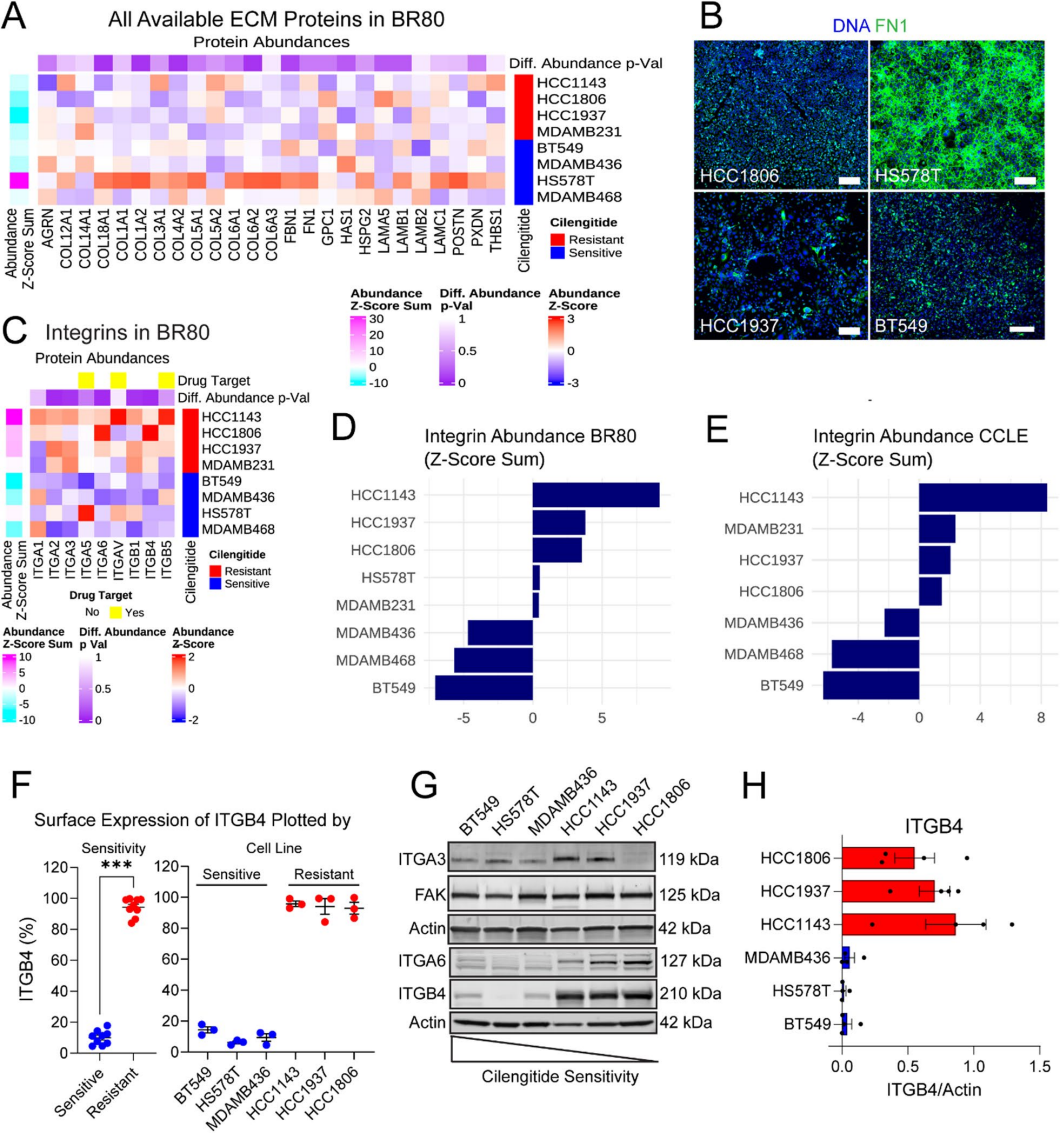
Proteomic characterization of TNBC cell lines used in drug screen. **A**. A heatmap shows proteomics data of ECM protein expression in resistant (red) and sensitive (blue) TNBC cell lines. **B**. Immunofluorescence of FN1 (green) counterstained with DAPI (blue) in resistant (HCC1806, HCC1937) and sensitive (HS578T, BT549) cell lines. Scale bar = 200 µm. **C**. Integrin protein expression in resistant (red) and sensitive (blue) breast cancer cell lines from the BR80 proteomics dataset. The integrins targeted by cilengitide are labeled with yellow boxes. The p-value (unadjusted Wilcoxon signed rank) of differential abundance between resistant and sensitive lines is shown, with more significant p-values in darker purple. **D**. The z-score values from (**C**) were summed across rows for the available integrins and plotted to show relative integrin abundance across the cell lines. **E**. The z-scores from integrins in the CCLE were summed across cell lines and plotted as in **D**. **F**. Surface-expressed ITGB4 was assessed using flow cytometry and percentages of positive cells were quantified for each cell line, then plotted by sensitivity to cilengitide (left) or by cell line (right). A significant difference was found (t-test) between the sensitive and resistant lines (p < 0.001). The mean with S.E.M. is shown. **G**. Lysates were made from untreated cells (arranged from most to least sensitive; see triangle), and subjected to immunoblotting for several integrins, with beta-actin serving as a loading control. ITGA6 and ITGB4 were probed on the same membrane as ITGAV and ITGB3 in Fig. 3D (the same actin band appears in both figures). ITGA3 and FAK were run on a parallel blot and have a separate actin loading control. **H**. Three independent cell protein lysates from each cell line were quantified for ITGB4 and normalized to beta-actin. Resistant lines are colored in red, while sensitive lines are blue. The mean with S.E.M. is shown

Given the implication of integrin-mediated cell adhesion as the differentiator in ITGAV inhibition response, we probed the BR80 proteomics dataset, specifically examining the integrins and ECM proteins. The sensitive line HS578T showed marked upregulation of many ECM proteins (Fig. 4A), especially FN1, which was confirmed using immunocytochemistry (Fig. 4B). However, no other sensitive line exhibited this phenotype, making it unlikely to explain cell response to cilengitide. Overall, the resistant lines had a tendency toward lower abundance of ECM proteins compared to sensitive lines (Fig. 4A). A more consistent sensitive-resistant line difference was observed for integrin expression. The resistant lines expressed more integrins and at higher levels than the sensitive lines (Fig. 4C–E), with many of the integrins trending towards a significantly higher expression in resistant lines compared to sensitive lines. In addition to the earlier-noted ITGA6 and ITGB4, which were significantly upregulated in resistant lines, ITGA2, ITGA3, and ITGB1 trended towards significantly higher expression in resistant lines (Fig. 4C). The CCLE dataset also showed increased integrin abundance in resistant lines (Supp. Figure 3A). Flow cytometry was used to validate significantly higher cell surface ITGB4 expression in resistant, though not sensitive lines (Fig. 4F). Additionally, we performed immunoblotting of cell lysates for several integrins as well as the focal adhesion kinase (FAK) which is involved in mediating cell attachment through integrins [41–43]. In our blotting experiments, we found that FAK was consistently abundant in all cell lines (Fig. 4G, Supp. Figure 3B). There was an apparent trend towards ITGA6 upregulation in resistant lines though the increase in ITGA3 was less clear in our immunoblots than it was in proteomics (Fig. 4G, Supp. Figure 3B); in contrast, ITGB4 was clearly higher in resistant lines (Fig. 4G–H), consistent with the proteomics data. We additionally visualized ITGA6 and ITGB4 across the cell lines to confirm anticipated localization of these integrins to sites of cell adhesion (Supp. Figure 3C).

We summarized our proteomics findings in graphs (Supp. Figure 4A) showing sensitive and resistant cell line integrin abundance in the context of their integrin binding partners and ECM proteins. This approach highlighted a few key differences in these two groups of cells. First, the ITGAV and ITGB5 monomers were more abundant in the sensitive lines compared to resistant lines, but only resistant lines showed a positive correlation between these proteins, suggesting a coordinated expression of these proteins that would result in increased dimer presence. The increased presence of this secondary cilengitide target on resistant cells could either represent a vulnerability because it would enable targeting by cilengitide, or a resistance mechanism because of the increased ratio of integrin target to cilengitide molecule. Given the cell phenotype observed, the latter possibility may be more likely. Second, ITGA6 and ITGB1 are positively correlated in lines sensitive to ITGAV inhibition mediated by cilengitide and GLPG0187, but not in the resistant lines, suggesting this pair may be a key laminin-binding mechanism in sensitive cells while resistant lines may rely more heavily on the ITGA6:ITGB4 integrin pair for laminin binding. However, ITGB1 abundance is lower in the sensitive lines compared to resistant lines, suggesting that it would be less effective at mediating adhesion to laminin than ITGA6:ITGB4 in the resistant lines. Thus, the sensitive lines would not have an alternative integrin-mediated attachment in the presence of ITGAV inhibition while resistant cells would, based on the presence of integrin monomer abundance and their correlations in the cell lines.

To explore the possibility of ITGA6:ITGB4 dimers representing a compensatory mechanism in the face of ITGAV blockade, we examined the correlation of protein abundance from the BR80 in complexes from the CORUM database that include these two integrins together with their ECM binding partner, laminin [44]. We reasoned that cells utilizing these specific complexes would express the components in a coordinated fashion. We found that pairs of ITGA6:ITGB4 complex members were more highly positively correlated using protein abundance in the BR80 across the ITGA6:ITGB4:Laminin 10/12 complex in resistant cell lines than in sensitive lines (Supp. Figure 4B), suggesting this complex is present and active in the resistant, but not sensitive, lines. Thus, different integrin and ECM repertoires might form the basis of resistant line continued attachment in the presence of cilengitide or GLPG0187.

Given the high expression of ITGA6, ITGA3, and ITGB4 in resistant cells relative to sensitive cells, we performed knockdown experiments with siRNAs targeting each of these integrins to determine their role in mediating cilengitide resistance. We did not observe increased sensitivity in resistant cells with knockdown of any of the integrins or in the presence of a blocking antibody to ITGB4 (Supp. Figure 5). While select knockdown of integrins failed to sensitize resistant cells to cilengitide, we cannot rule out the possibility that integrin crosstalk (e.g., a shift of an integrin monomer pairing with an alternative partner when another is knocked down [17, 45, 46] or compensatory upregulation [47, 48]) was the cause of persistent cilengitide resistance in the context of ITGA6, ITGA3, and ITGB4 knockdown.

### Both laminin and fibronectin can confer cilengitide resistance to sensitive cells

**Fig. 5:**
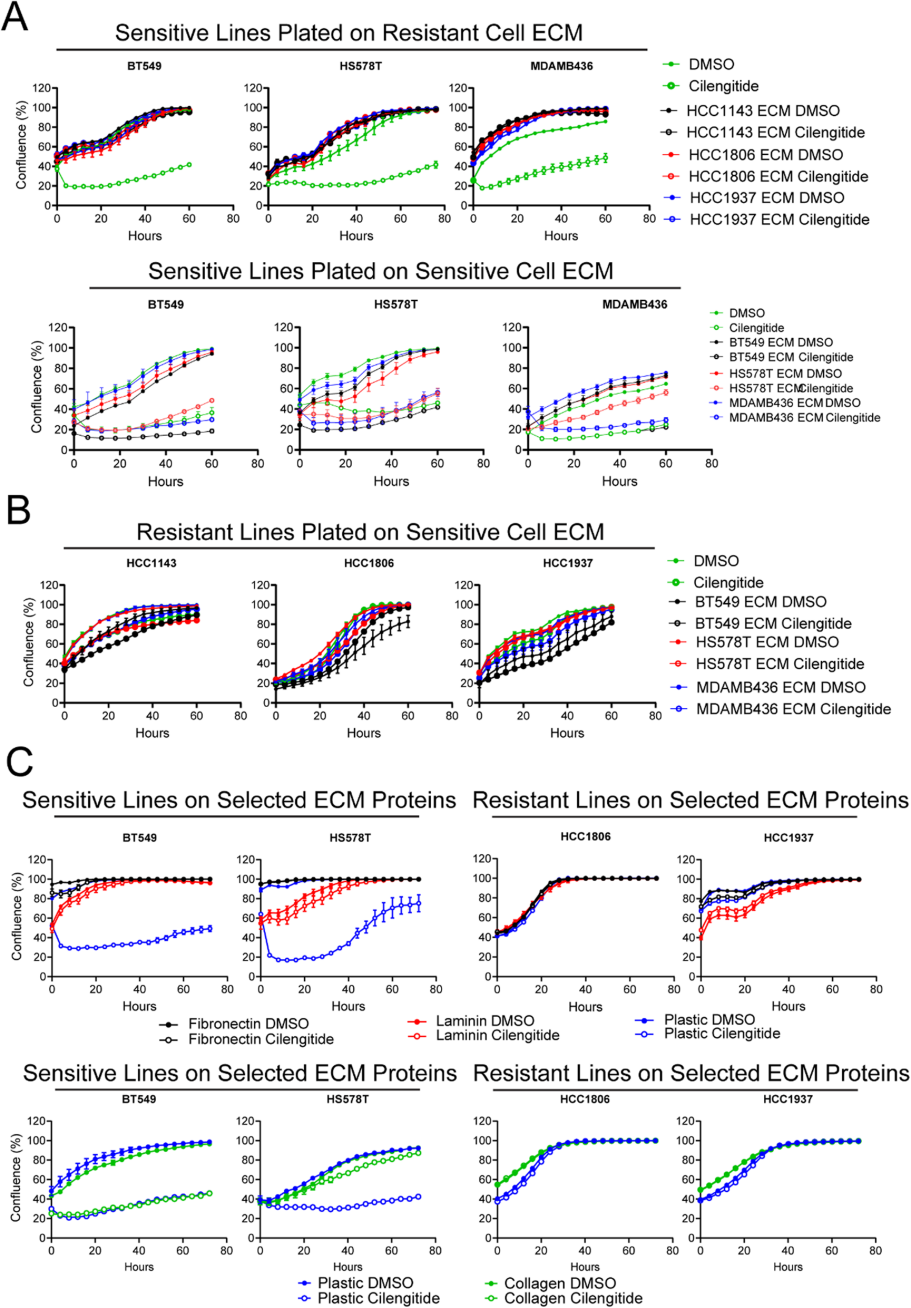
Cilengitide sensitivity can be modulated by ECM. **A**. Sensitive lines were plated in triplicate on ECM deposited by resistant cells (top row; HCC1143 ECM in black, HCC1806 ECM in red, HCC1937 ECM in blue) or sensitive cells (bottom row; BT549 ECM in black, HS578T ECM in red, MDAMB436 ECM in blue). Cells were then treated with DMSO (closed circle) or 5 μM cilengitide (open circle) and their confluence was monitored at 4 h intervals for 60 h. For comparison, cells were also plated on untreated plastic (green curves). **B**. Resistant lines were plated on ECM deposited by sensitive cells (bottom row; BT549 ECM in black, HS578T ECM in red, MDAMB436 ECM in blue), treated with DMSO or 5 μM cilengitide, and their confluence was monitored at 4 h intervals for 72 h. For comparison, cells were also plated on untreated plastic (green curves). **C**. Sensitive lines BT549 and HS578T and resistant lines HCC1806 and HCC1937 were plated in triplicate on fibronectin (black), laminin (red), collagen I (green), or plastic (blue), treated with DMSO or 5 μM cilengitide, and their confluence was measured at 4 h intervals for 72 h. In all cases, a representative experiment is shown of 3 (**A** & **B**) or 2 (**C**) biological replicates

The targets of cilengitide, integrin dimers ITGAV:ITGB3 and ITGAV:ITGB5, bind RGD motifs found in ECM components such as fibronectin, vitronectin, and fibrinogen [12, 13]. Collagen and laminin are bound by other groups of integrins, including many integrin dimers formed by the ITGB1 subunit and the ITGA6:ITGB4 dimer [49], which are not targets of cilengitide. Thus, cilengitide sensitivity of a cell might be impacted by the available ECM substrates. To explore this possibility, we plated resistant cell lines and allowed them to deposit matrix for 2–3 days under normal growth conditions before decellularizing the plates and seeding sensitive lines on the deposited ECM proteins. The sensitive lines were allowed to adhere overnight then treated with cilengitide. The resistant-cell ECM proteins rendered sensitive lines resistant to cilengitide (Fig. 5A, top row; Supp. Figure 6A). To test whether increased matrix protein deposition may be the cause of the newly acquired resistance, each sensitive line was plated on ECM proteins deposited from each sensitive line (i.e., BT549 was plated on its own ECM as well as HS578T ECM, and MDAMB436 ECM). We found that sensitive-line ECM proteins did not confer cilengitide resistance, with the exception of the matrix proteins from line HS578T for MDAMB436 cells (Fig. 5A, bottom row; Supp. Figure 6A). Also, the ECM of sensitive lines did not confer sensitivity to resistant lines (Fig. 5B; Supp. Figure 6A). These results suggest that the ECM deposited by cancer cells can dictate response to cilengitide.

While plating cells on different ECM deposits, we noted that the ECM deposited by the resistant cell lines induced altered cell morphology in some sensitive lines (Supp. Figure 6B), though not the other way around (Supp. Figure 6C). Given that the resistant ECM converted sensitive cells to resistant cells, we aimed to characterize these morphological changes to see if they predicted cilengitide sensitivity. For example, cilengitide-sensitive MDAMB436 cells acquired a flattened morphology on HCC1806 and HCC1937 matrices, compared to its morphology on all three sensitive cell matrices, where it formed round cells with multiple projections (Supp. Figure 6B). Indeed, the matrix from cilengitide-resistant HCC1806 caused spreading in all three sensitive lines. However, while cilengitide-resistant HCC1937 ECM conferred resistance to all sensitive lines, it only induced morphological changes for BT549 and MDAMB436. Similarly, cilengitide-resistant HCC1143 ECM rendered all three sensitive lines resistant, but only HS578T acquired a different appearance. Thus, cell morphology changes were not always predictive of cilengitide response.

To better understand which ECM proteins may confer resistance to cells, we plated cells on laminin, collagen, or fibronectin. As was seen on cell line ECM, there were morphological shifts of sensitive cells on the different ECM substrates. Resistant lines also exhibited some morphology changes, though cilengitide-resistant HCC1806 remained largely unchanged regardless of ECM protein (Supp. Figure 6D). All tested lines were resistant to cilengitide treatment when plated on laminin or fibronectin (Fig. 5C). Collagen I also conferred resistance, except to cell line BT549 (Fig. 5C). Taken together, these results show that multiple ECM proteins can modulate cilengitide sensitivity but are only a part of what makes a cell sensitive to cilengitide.

### Luminal breast cancer cells with lower integrin expression are sensitive to cilengitide

**Fig. 6:**
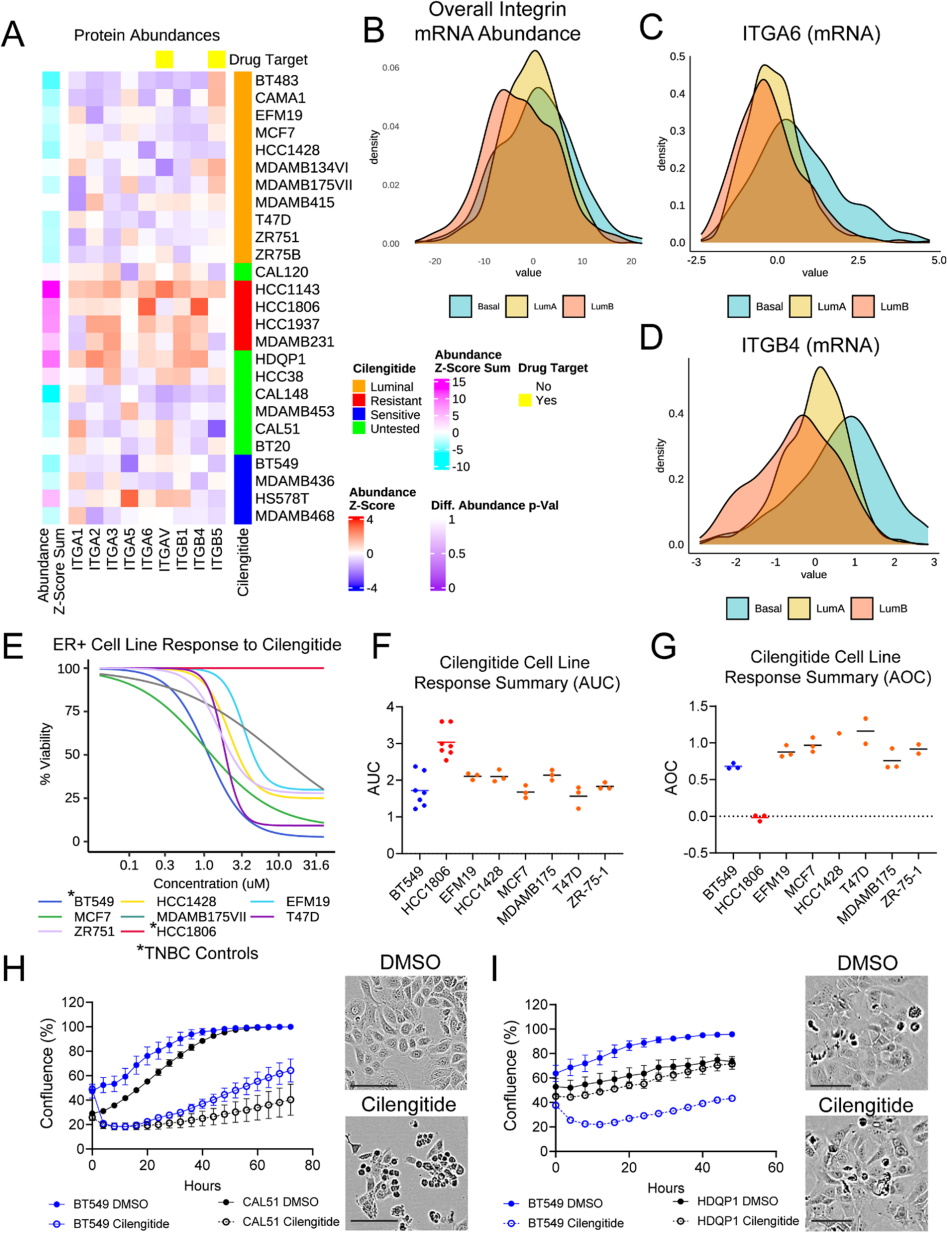
Breast cancer cell lines expressing lower levels of integrins are more sensitive to cilengitide. **A**. A heatmap of integrin protein expression in luminal (orange) and TNBC breast cancer cell lines from the BR80 proteomics dataset is shown. Known resistant lines are marked in red, sensitive lines in blue and lines with untested cilengitide response were marked in green. Z-score values were summed across rows to calculate overall abundance for the available integrins (magenta higher and cyan lower abundance). **B**–**D**. Density plots show the sum of overall integrin expression scores (**B**), or ITGA6 (**C**) or ITGB4 (**D**) protein abundance for breast cancer patient data, plotted by subtype (Basal = blue, Luminal A = yellow, Luminal B = red), from the METABRIC dataset. **E**. Luminal cell line dose response curves were fitted based on a 9-point titration of cilengitide. One of 3 representative experiments is shown. HCC1806 and BT549 are included for comparison. **F**. The AUC values for luminal lines (orange symbols) were compared with AUC values of a resistant (HCC1806; red) and a sensitive (BT549; blue) cell line. **G**. The AOC values for luminal lines (orange symbols) were compared with AOC values of a resistant (HCC1806; red) and a sensitive (BT549; blue) cell line. **H**. CAL51 (black) and known-sensitive line BT549 (blue) were plated in triplicate, treated with DMSO (circle, solid line) or 5 μM cilengitide (open circle, dotted line) and their confluence was monitored at 4 h intervals for 72 h. One of three representative experiments is presented. CAL51 cell morphology 72 h after treatment with cilengitide or DMSO is shown. **I**. HDQP1 (black) and known-sensitive line BT549 (blue) were plated in triplicate, treated with DMSO (circle, solid line) or 5 μM cilengitide (open circle, dotted line) and their confluence was monitored at 4 h intervals for 72 h. One of three representative experiments is presented. HDQP1 cell morphology 72 h after treatment with cilengitide or DMSO is shown. Scale bars = 100 μm

Considering the results from our integrin knockdown experiments showing that integrin-abundant lines are not sensitized to cilengitide, and the observation that cilengitide resistant cells express higher levels of multiple integrins, we sought to test whether cell lines expressing lower overall integrin levels might be sensitive to cilengitide. We reasoned that such integrin low cells would lack compensatory binding from integrins not targeted by cilengitide. Thus, when the dimers targeted by cilengitide (e.g., ITGAV:ITGB3) would be inhibited, this type of cell would be dislodged from the matrix. Previous studies in the normal mammary epithelium have shown that basal breast epithelial cells express more integrins, including the ITGA6:ITGB4 dimer, than luminal epithelial cells [45, 50]. Hence, we checked the integrin repertoire expressed by several luminal lines in the BR80 dataset. In line with the biology of the normal mammary epithelium, we found that luminal breast cancer cell lines expressed lower abundance of integrins than TNBC lines, with an especially striking difference between luminal and resistant TNBC lines (Fig. 6A, Supp. Figure 7A–B). Also, in the BR80, we noted additional integrin-low TNBC lines (e.g., CAL51, CAL148) and integrin-high TNBC lines (e.g., HDQP1, HCC38). The overall elevated integrin expression in basal tumors over luminal tumors was confirmed in patient data from the TCGA (Fig. 6B). Basal tumors also tended to express more ITGA6 and ITGB4 than luminal tumors, similar to our observations made in the cell lines (Fig. 6C and D). By employing the same approaches used with TNBC cell lines, we confirmed that the six tested luminal lines (EFM19, HCC1428, MCF7, MDAMB175VII, T47D, and ZR-75–1) exhibited the hallmarks of cilengitide sensitive lines, such as low AUC values, absent or suppressed growth, and cell detachment in the presence of cilengitide (Fig. 6E–G, Supp. Figure 7C). Interestingly, fibronectin rescued proliferation in four of the six lines and laminin rescued in only two of the six lines, in contrast to the universal rescue seen with both matrix proteins in TNBC lines (Supp. Figure 7D), possibly because different integrin repertoires will result in differential pathway activation even on the same substrate [51]. These data support the hypothesis that decreased overall integrin expression indicates increased cilengitide sensitivity and suggest that, in addition to utility in a subset of TNBC patients, cilengitide, or other ITGAV inhibitors, may be effective in luminal breast cancers as a whole.

**Fig. 7:**
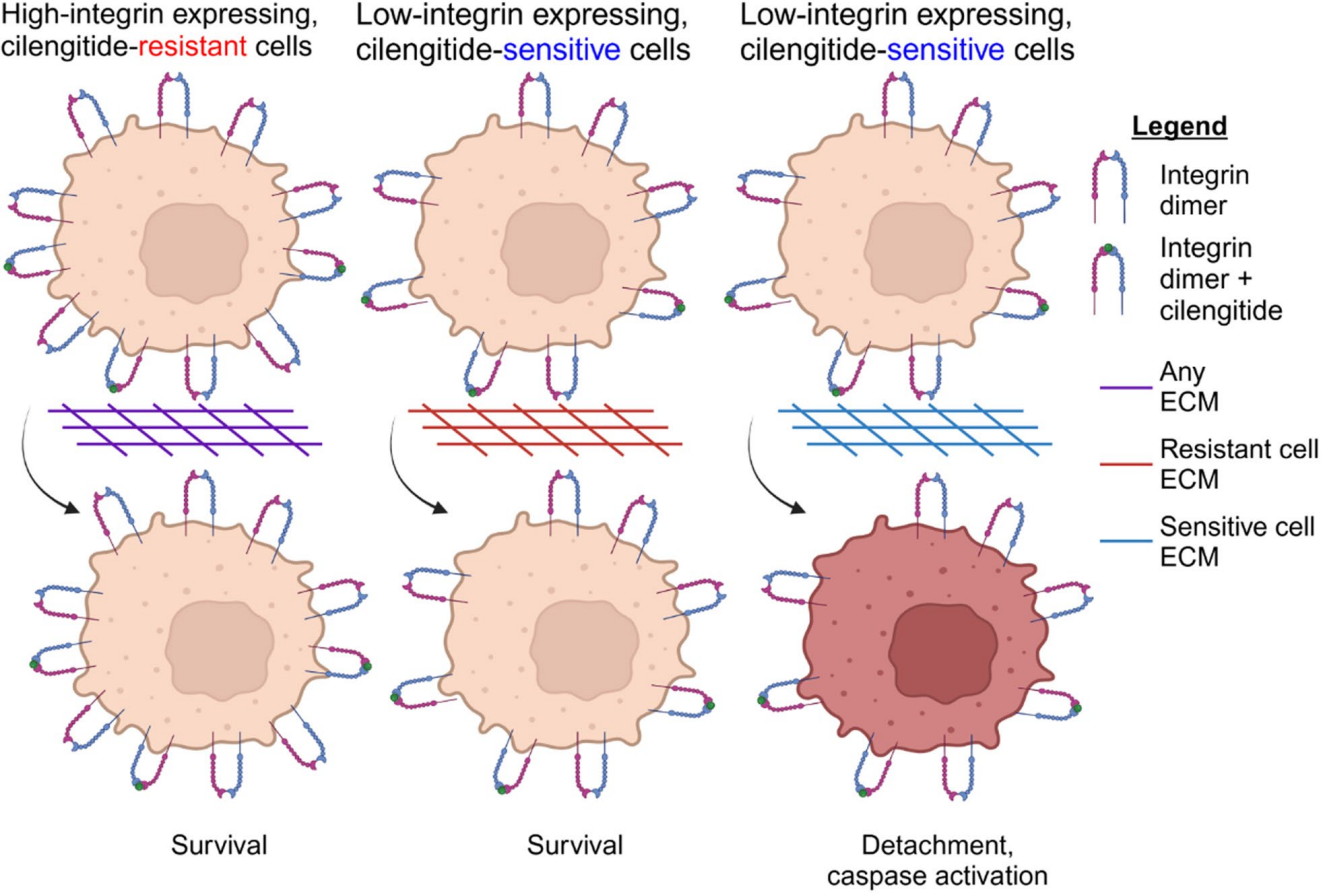
Integrin and ECM protein interactions differ between cilengitide-resistant and cilengitide-sensitive TNBC lines. A model summarizes the findings that high integrin abundance results in cell survival during cilengitide treatment, regardless of ECM proteins present. Meanwhile, in cells expressing fewer integrins, a complementary ECM can promote cell survival

Our collective findings suggested that overall integrin expression may predict cilengitide sensitivity better than a single integrin. To further test the potential of using integrin abundance, we sought to perform drug testing in a set of TNBC cells that were not included in our original set of lines. Of the TNBC cell lines not yet tested, we found that the integrin-low line CAL51 (Fig. 6A) showed reduced growth and cell rounding in the presence of cilengitide similar to the sensitive line BT549 (Fig. 6H). Meanwhile, integrin-high HDQP1 (Fig. 6A) cells proliferated in the presence of drug and their morphology was unchanged (Fig. 6I). Thus, our hypothesis that integrin-abundant cell lines would be resistant to cilengitide (Fig. 7) was confirmed, demonstrating that we were able to accurately predict in vitro TNBC cilengitide sensitivity based on overall integrin expression.

## Discussion

Due to a lack of known targetable vulnerabilities in TNBC, patients with this disease have few treatment options. To address this need, we executed a drug screen on 45 compounds targeting TNBC vulnerabilities based on analysis of the Broad DepMap to identify drug compounds with an associated exploratory biomarker that could be used for patient stratification by potential treatment benefit. Several studies have reported that the use of biomarkers, including exploratory biomarkers [52], increases clinical trial success rates across clinical trials in various cancers (gastric, breast cancer, melanoma and NSCLC) [52–55]. We found that the integrin inhibitor cilengitide showed a variable response across TNBC cell lines, making it amenable to biomarker identification (Fig. 2). Furthermore, the mixed performance of ITGAV inhibitors in clinical trials [19, 21–24] suggests that their clinical application would benefit from a biomarker.

We paired large-scale mass-spectrometry proteomics data collected on the BR80 and CCLE cell line collections with in-house drug screening for a systematic analysis to expand on previous experimental work studying TNBC, integrins, and cilengitide sensitivity [16, 17]. We found that the main differentiating features between sensitive and resistant lines was upregulation of proteins in cellular adhesion pathways, in particular the integrins ITGA6 and ITGB4, in resistant lines compared to sensitive lines. In contrast, we were not able to find a relationship between cilengitide sensitivity and cilengitide target integrins ITGAV, ITGB3, or ITGB5, contrary to previous observations in breast cancer cell lines [16] and glioblastoma [38]. Based on our studies in TNBC, we propose the examination of overall integrin expression to predict cilengitide response (Fig. 7).

The integrin family of proteins is highly redundant [56] and exhibits extensive crosstalk [46]. Thus, the inhibition of ITGAV:ITGB3 (i.e., αvβ3) and ITGAV:ITGB5 (i.e., αvβ5) by cilengitide does not represent a meaningful reduction in cell adhesions for a cell expressing many alternative integrin pairs, as shown in Fig. 6E–I. We draw this conclusion from our observations (1) that single integrin knockdown in resistant lines is not sufficient to induce cell detachment or cilengitide sensitization (Supp. Figure 5); (2) that the number of apparent attachments in the resistant lines is greater than in sensitive lines, based on the overall overexpression of the integrins (Fig. 6A) and their correlations (Supp. Figure 4A & B); and 3) that cilengitide sensitivity in normal culture conditions can be predicted based on overall integrin expression (Fig. 6H–I).

Multigene signatures can accurately discriminate between breast cancer subtypes [57–60], predict responsiveness to chemotherapy [61], and have successfully been used for clinical trial eligibility criteria with TNBC and prostate cancer (3 and 300 gene signatures, respectively) [62, 63]. A signature derived from overall integrin proteomic abundances may be a useful tool to discriminate between vulnerability or resistance to integrin inhibition. Clinically, a lot of focus has been on routine analysis of somatic mutations to help guide patient care [64, 65] with efforts to expand this to RNA sequencing (e.g., the NCI COMPASS program), but analogous high-throughput clinical proteomics lag. Large-scale efforts, such as Pi-Hub (https://www.pi-hub.org.cn/) and the European Proteomics Infrastructure consortium [66], that seek to perform proteomics on patient samples at scale will further the clinical use of proteomics and our understanding human disease. RNA expression is not necessarily indicative of protein abundance [67, 68], with the average protein–RNA correlation at 0.4–0.6 [69], suggesting that proteomic characterization may reveal distinct features not seen at the RNA level. With further algorithmic deconvolution, such proteomics may enable assessment of the protein complexes present [70].

Integrins mediate attachment to the ECM through two structures: focal adhesions or hemidesmosomes. ITGA6 and ITGB4 together with plectin, BP230, and BP180 form the hemidesmosomes which attach to laminin [71]. These two attachment complexes arrange specific arrays relative to each other, each promoting the other’s efficient formation, and together enable keratinocyte migration [72]. The loss of ITGB4 in keratinocytes has also been shown to promote focal adhesion formation and increase cell spreading [73]. Thus, crosstalk between these different cell adhesion complexes impacts each other’s formation. Based on protein abundance correlation, hemidesmosomes are likely more prevalent in the resistant lines compared to the sensitive lines (Supp. Figure 4B). However, previous studies have shown that hemidesmosomes are lost over the course of breast cancer progression in vivo and suggest that cell lines may not consistently form this structure [74]. Nevertheless, ITGA6:ITGB4 expression has been shown to promote the surface expression of focal adhesion integrins in keratinocytes [75], another example of hemidesmosome-focal adhesion crosstalk. This observation supports the idea that a similar mechanism may explain the high overall integrin expression in cilengitide-resistant cells which were significantly higher than sensitive cells for ITGA6 and ITGB4 protein abundance (Supp. Table 5, Fig. 4C–H).

The observation that ECM composition can influence cilengitide response highlights the need to understand cell response to integrin inhibition in different microenvironments: cancer cells will undergo extrinsic changes as they inhabit new niches and intrinsic changes as they continue to evolve. Hypoxic zones, which occur in TNBC tumors [76], are composed of high levels of FN1 and other RGD motif-containing proteins. The bone marrow also expresses high levels of FN1 [77, 78], and this environment appears to induce ITGB3 upregulation in metastasized breast cancer cells [78]. If this upregulation of ITGB3 in the context of high FN1 occurs together with downregulation of laminin-binding integrins like ITGA6, ITGB1, and ITGB4, it may represent a cell state that would be similar to the HS578T cell line (i.e., high for ITGB3 (Fig. 3), low for other integrins (Fig. 4)) and thus, primed for response to cilengitide. Interestingly, a preclinical study in rats suggested that cilengitide can slow the process of bone colonization by breast cancer metastases [79]. Indeed, several in vivo studies using cilengitide have shown a benefit to using the drug in the context of glioma [80], pancreatic ductal adenocarcinoma [81], melanoma [82], and breast cancer [10, 83]. While most of these studies use a single model, a systematic examination of several models with differing in vitro responses would provide additional insight on how cilengitide would perform in different tumor microenvironments in vivo and is out of the scope of this paper focused on TNBC.

Although the focus of this work is TNBC, our results suggested examination of luminal breast cancer response to cilengitide because of the parallels between breast epithelial cell biology and breast cancers. Just as luminal epithelial cells express fewer integrins than basal cells [45, 50], TNBC cell lines, which represent basal tumors, express more integrins than ER + lines (Fig. 6A). We then found that all six of the ER + tested lines were sensitive to cilengitide, and more than half remained sensitive when plated on fibronectin. This finding of ER + breast cancer line sensitivity raises the intriguing question of whether cilengitide may be effective in the endocrine therapy-resistance stage of disease [84]. ITGA6 may be upregulated with the acquisition of tamoxifen resistance in cell lines, or following relapse in patients [85]. Other work in luminal cell lines showed that ITGB1 can be upregulated in tamoxifen-resistant cells [86]. While these studies focused on single integrins, they provide evidence of integrin changes in the context of luminal breast cancer and motivate follow-up studies that may establish ER therapy-resistant cell integrin profiles.

In addition to our observations on integrin and ECM expression and their related roles in cilengitide response, we provide a useful resource for exploring additional treatment options for TNBC. Our drug screen dataset includes 45 compounds (Supp. Table 6), 18 of which were previously unscreened, across eight cell lines, including African American cell lines, which represent an under-studied population. Fourteen of the tested drugs showed little or no effect on cell viability despite having been identified as genetic dependencies, likely because drug dosing involves using small molecules with varied potencies and targeting mechanisms at different time scales than genetic knock-out. Still, six drugs produced high AUC values across all tested lines indicating greater drug sensitivity and suggesting that they could be potent drugs to use in TNBC. Thus, datasets like the DepMap that inform on gene dependencies are valuable tools: they unveil important pathways and processes involved in cell viability. Follow up experiments like those described here are necessary to determine which dependencies translate to drug sensitivity. The compounds identified in this screen with a wide range of responses across cell lines represent strong candidates to pursue in future biomarker studies like the ones performed here.

Although we have identified cilengitide as an effective cytotoxic drug for a subset of TNBC, and possibly breast cancer more broadly, there are limitations to our study that could be expanded on in future work. First, because our drug screen included very diverse compounds, it was conducted under standard cell culture conditions, which include fetal bovine serum. This component of the media can lead to vitronectin coating of the plate, which promotes ITGAV:ITGB3/ITGAV:ITGB5 binding. Second, we focused on 2D studies, keeping in vivo experiments for future work. The optimal design of this future work would include multiple cell line models of differing cilengitide sensitivity so that the in vitro cilengitide response could be related to the in vivo response, and changes that may occur to the cell lines when engrafted in a host could be thoroughly examined. Finally, while our ECM experiments aim to better elucidate how cilengitide would perform in vivo, they were not performed with ECM deposited by stromal cells, potentially yielding a matrix composition that is different from physiological conditions. Still, we tested ECM proteins that are highly expressed in the breast tissue (laminin and collagen I) to better assess cilengitide sensitivity in a related environment [87].

Our results and the works of others (e.g., Haddad et al.) suggest that there is more to be understood regarding integrin inhibition as a therapeutic target in cancer than was determined by the results of the CENTRIC trial [18]. Collectively, this work has provided further characterization of several frequently used TNBC cell lines in the drug response and cell adhesion fields of study. As other integrin-targeting therapies are developed [88] and patients are enrolled in new trials (NCT05085548 testing an αVβ3 inhibitor, NCT06603844 testing an αVβ8 inhibitor), the observations herein may provide insight into strategies for the use of this class of drugs.

## Methods

### Cell culture

All TNBC cell lines, T47D, ZR-750-1, and EFM19 were cultured in RPMI supplemented with penicillin/streptomycin and 10% fetal bovine serum. MDAMB175VII, MCF7, and KPL1 were cultured in DMEM also supplemented with penicillin/streptomycin and 10% FBS. STR analysis was performed to confirm cell line identity and mycoplasma testing showed no contamination of these lines. Fluorescent caspase 3/7 dye was acquired from Sartorius and used at a 1:1000 dilution. For growth on laminin and fibronectin, plates were coated at 4 μg/cm^2^. Anti-ITGB4 antibody (Millipore Cat# MAB2059, RRID: AB_94526) was used in culture at 10 μg/ml. The drug GLPG0187 (MedChemExpress, Cat. HY-100506) was dissolved in DMSO used at 2.5 µM. For all 96 well experiments, cells were plated at a density of 10,000 cells/well, with a minimum of 3 technical replicates per experiment, and imaged in an Incucyte Zoom or Incucyte S3 (Sartorius) using a 10 × objective at the time intervals specified.

### Drug screening

To test cell line sensitivity to our selected compounds, we performed several rounds of screening, with drugs showing differential sensitivity across cell lines advancing to subsequent rounds of replication. Drugs that were universally toxic or showed no effect were dropped after the first replicate. All screens were performed as follows. To generate dose response curves, cells were grown to 80–90% confluence, then counted and seeded in 384 well plates at 750–1000 cells/30 μl of growth media per well using a Thermo Multidrop Combi. One cell line was seeded per 384-well plate. An additional 20 μl of growth media were added to bring the total volume to 50 μl per well. Twenty-four hours after plating, cells were dosed with drugs (Supp. Table 1) using a HP D300e (Hewlett Packard). The range of doses used for each drug can be found in Supp. Table 6. Each dose-cell line combination had two technical replicates. Treatment lasted for 72 h, then cells were washed with 1 × PBS, fixed and stained with a PBS-based solution of 3.7% paraformaldehyde and Hoechst (5 μg/ml), and transferred to 0.1% sodium azide in PBS for storage. All liquid handling was done using a Thermo Multidrop Combi. The plates were imaged using an Acumen eX3/HCI (TTPLabTech).

### Flow cytometry

Cell suspensions of each TNBC cell line were stained for 9 min at RT with Zombie Green Fixable Viability Dye (Biolegend 423,111) according to manufacturer’s instructions. Suspensions were then washed twice with PBS + 2% heat-inactivated FBS (hereafter, cell-staining medium [CSM]) and then fixed with 1.6% PFA (diluted 1:10 in PBS from 16% PFA Electron Microscopy Sciences 15,710) for 30 min at RT. Following two more washes with CSM, cells were stored overnight at 4 degrees. Half of the cells from each cell line were then stained for 25 min at RT with PE-conjugated anti-CD104 (ITGB4) antibody (Biolegend 327,807) and APC-conjugated anti-CD51/61 antibody (Biolegend 304,415), both at a dilution of 1:100 in CSM. Concurrently, the remaining cells from a given cell line were maintained in CSM without antibody for an unstained control sample. Cells were washed twice with CSM and then analyzed using a FACSCalibur (BD Biosciences). In Flowjo, forward scatter, side scatter, and the viability dye were used to remove doublets, dead cells, debris, and other low-quality events. For each sample, 30,000 events were collected, and at least 20,000 cells were analyzed following quality control. Unstained controls for each cell line were used to gate for both CD104 and CD51/61, and the fraction of each cell line positive for each integrin was calculated. This experiment was performed in triplicate.

### Immunocytochemistry

Cells were plated at equal densities on glass coverslips. Coverslips were fixed the following day in 4% Paraformaldehyde for 10 min and washed with 1X PBS 3 times. Cells were permeabilized in 0.1% PBS-Tween for 5 min, washed 3 times in 1X PBS, and then blocked with 1% BSA/10% normal goat serum/0.3 M glycine in 0.1% PBS-Tween for 1 h. The cells were then incubated overnight at 4 °C with the FN1 Fibronectin antibody (Abcam Cat# ab198933, RRID: AB_2728807; dilution 1:200), ITGAV:ITGB3 antibody (Abcam Cat# ab190147, RRID: AB_2925190; dilution 5 µg/ml (1:200 dilution with blocking buffer)), ITGB4 (Cell Signaling Technology Cat# 14,803, RRID: AB_2798620; dilution 1:100), or ITGA6 (BD Bioscience Cat# 555,734, RRID: AB_2296273; dilution 1:100) and counterstained with DAPI. Coverslips were washed 3 × with 1X PBS and mounted on Fisherbrand Colorfrost plus microscope slides (catalog: 12–550-16 Fisherbrand) using Prolong Gold anti-fade reagent (P36934 Invitrogen). Images were acquired on a Yokogawa spinning disk confocal on an inverted Nikon Ti fluorescence microscope, with consistent exposure times across samples.

### Immunoblotting

Proteins were extracted using the Cell Extraction Buffer (FNN0011 Thermofisher) according to the manufacturer’s instructions, normalized using Bio-Rad DC™ Protein Assay Kit I (5,000,111 Bio-Rad), and 20 μg were loaded onto and run on a pH 3–10 Criterion™ IEF Gel, 12 + 2 well (3,450,071 Bio-Rad) for immunoblotting. All blocking steps were performed using Li-Cor Intercept PBS Blocking Buffer (927–70,001 Li-Cor Biosciences) for 1 h at room temperature, and primary antibody incubations were performed overnight at 4 °C. The membranes were activated with Immobilon Classico Western HRP substrate, 500 mL (WBLUC0500 Millipore-Sigma), for 5 min. Extracts (20 μg) were subjected to analysis with antibodies for FAK (Cell Signaling Technology Cat# 13,009, RRID: AB_2798086; dilution 1:2000), beta-Actin (Sigma-Aldrich Cat# A1978, RRID: AB_476692; dilution 1:5000), Integrin B4 (Cell Signaling Technology Cat# 14,803, RRID: AB_2798620; dilution 1:1000), Integrin Alpha 6 (Abcam Cat# ab235905, RRID:AB_2925230; dilution 1:500), Integrin Beta3 (Cell Signaling Technology Cat# 13166t, RRID: AB_2798136; dilution 1:500), Integrin Alpha V (Cell Signaling Technology Cat# 4711 s, RRID:AB_2128178 dilution 1:500), and Integrin A3 (Abcam Cat# ab242196, RRID: AB_2920907; dilution 1:500). Antibodies were detected with HRP-Linked Anti-Mouse (Cell Signaling Technology Cat# 7076, RRID: AB_330924 dilution 1:10,000), HRP-Linked Anti-Rabbit (Cell Signaling Technology Cat# 7074, RRID: AB_2099233; dilution 1:10,000), IRDye Anti-Rabbit 800CW (LI-COR Biosciences Cat# 926–32,211, RRID: AB_621843; dilution 1:4000), and IRDye Anti-Mouse 680LT (LI-COR Biosciences Cat# 926–68,020, RRID: AB_10706161; dilution 1:4000). The membrane was exposed on film in the dark room, and then the film was scanned using an Epson scanner (EPSON Perfection 4990 PHOTO), or imaged using Li-Cor Odyssey Clx Imager. To visualize multiple antibodies on a single membrane, the membranes were stripped for 15 min at room temperature using Pierce Restore™ PLUS Western Blot Stripping Buffer 500 mL (46,430 Life Technology (through VWR)) and blocked before incubating with additional primary antibodies.

### Knock-down

Cells were allowed to reach ~ 40% confluence before transfection with 25 nM shRNA smartpools (Horizon) using RNAiMax (ThermoFisher), following manufacturer’s instructions. Two to 3 days following transfection, cells were counted, plated in 96 well plates with 3 technical replicates per condition, and allowed to adhere overnight before treatment with DMSO or cilengitide (5 μM). Imaging was performed using an IncucyteZoom or IncucyteS3.

### Cell plating on deposited ECM

Cells were cultured 2–3 days in 96 well plates to allow ECM deposition. The ECM was then decellularized by treatment of cells with 20 mM NH_4_OH 0.5% Triton-x in PBS for 20 min at 37 C. Cell removal was confirmed by visual inspection. The plates were subjected to 3 washes with 1 × PBS to ensure no detergent was carried over. Cells plated on the decellularized ECM were given 8 h to adhere before treatment with cilengitide or DMSO and imaging using an IncucyteZoom or IncucyteS3 at 4 h intervals for 48 h. Three technical replicates were included per condition per experiment.

### Computational and data analysis

#### Fitting dose response (IC50, AUC, GR) and drug annotation

IC50 and AUC were calculated using the PharmacoGx R package (version 1.17.1). The drug potency (GR50) and drug efficiency (GRmax) were determined for each compound using the GRmetrics (version 1.12.2) package using R (version 3.6.2). Drug mechanisms were annotated using the signaling pathway category for each drug given on the Selleck Chemicals website. The authors also annotated the drugs with the rationale used for their selection largely based on dependencies or biological processes targeted by the drug.

#### Statistical analysis and visualizations

Statistics and genomic association with response was calculated using unpaired two-samples Wilcoxon tests were performed in R (3.6.2). Visualizations (i.e., density, boxplots, and heatmaps) were generated using the ggplot2 (version 3.3.6). Figure 7A was generated in BioRender. GraphPad Prism was used to generate all growth curves (i.e., confluence versus time), AUC and AOC column plots, and bar plots of gene dependencies.

#### Gene enrichment analysis

Fgsea package 1.12.0 was used for the enrichment analyses using Hallmark signatures together with selected KEGG and WikiPathway signatures involving ECM, focal adhesion, NOTCH signaling, as well Kohn EMT gene signatures from MSigDB [89]. The rank metric is as follows: sign(fold change of gene_i)* − log10(P_i) where the fold change is the ratio of the mean of the resistant cell lines versus the sensitive lines. The p-value (P_i) is taken from a t-test comparing resistant versus sensitive cell lines. With this rank metric, up-regulated genes with smaller p-values appear at the top of the list and down-regulated genes with smaller p-values at the bottom.

#### METABRIC expression

Log-transformed mRNA z-Scores compared to the expression distribution of all samples (Illumina HT-12 v3 microarray) were obtained from the cBioPortal datahub from the METABRIC study (N = 1906) https://github.com/cBioPortal/datahub/tree/master/public/brca_metabric/data_mrna_illumina_microarray_zscores_ref_diploid_samples.txt; previously named: data_mRNA_median_all_sample_Zscores.txt [90–94]. Classification of samples was taken from the CLAUDIN_SUBTYPE annotations provided.

#### Proteomics datasets

BR80 proteomics log2-scaled protein intensity values are used; this is a collection of breast cancer cell lines [38]. Additionally, protein abundance values for CCLE cell lines was obtained from Nusinow et al. for select cell lines. The values represent protein abundance ratios relative to the abundance of reference proteins in “bridge samples”; specific file used “Protein Quantitation (TSV Format)”: protein_quant_current_normalized.csv [40]. Since these data were already normalized, no further preprocessing was applied.

#### CORUM protein complexes

A list of protein complexes and involved subunits was obtained from the comprehensive resource of mammalian protein complexes (CORUM) database. CORUM defines protein complexes as a group of two or more proteins that physically interact and form a quaternary structure. Data was accessed from CORUM Version 3 on June 16, 2020 [95].

#### DisGeNET gene-disease associations

A list of gene-disease associations (for filtering to genes specific to TNBC) were obtained from the DisGeNet database version 7.0 [96]. Disease identifier (UMLS/CUI: C3539878) for “Triple negative breast cancer” was used as a search query term to return associated genes.

#### Broad DepMap (Project Achilles) CRISPR

Broad DepMap (Project Achilles) data (ceres_gene_effects_17Q2v2.csv) was used to determine dependencies of interest. The Broad DepMap project reports essentiality scores using the CERES algorithm. A lower CERES score indicates a higher likelihood that the gene of interest is essential in a given cell line. 0 indicates the gene is not essential and − 1 is comparable to the median of all pan-essential genes [97].

#### Retrieval FAKi sensitvity and comparison to cilengitide response

The Genomics of Drug Sensitivity in Cancer (GDSC) database was searched for “FAK” inhibitors revealing two inhibitors: GSK2256098C, PF-562271. The IC50 values for these two inhibitors in overlapping cell lines was extracted and correlated in against cilengitide mean IC50 values using Pearson's correlation test in the R programming language.

## Supplementary Information

Supplementary material 1.

Supplementary material 2.

Supplementary material 3.

Supplementary material 4.

Supplementary material 5.

Supplementary material 6.

Supplementary material 7.

Supplementary material 8.

## Data Availability

Data is provided within the manuscript or supplementary information files.
