# Supplementary material for: Cilengitide sensitivity is predicted by overall integrin expression in breast cancer
Source: Breast Cancer Res. 2024 Dec 20;26:187. doi: 10.1186/s13058-024-01942-2 (PMC11660856; doi:10.1186/s13058-024-01942-2)
Supplement: Supplementary file 7 — Supplementary material 7. [file 13058_2024_1942_MOESM7_ESM.docx]

**UNCROPPED IMMUNOBLOT IMAGES**

Figure 3 & 4.

ITGB3 (90) and ITGB4 (210) and actin


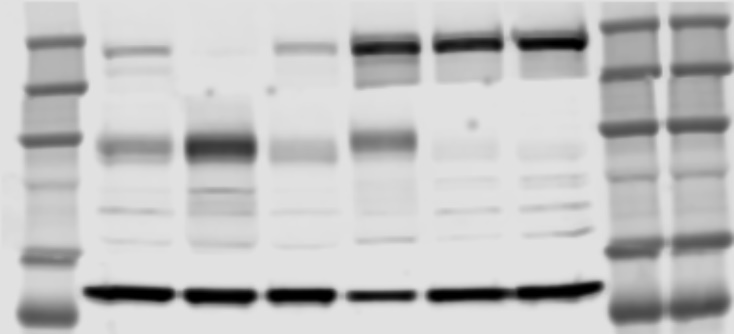


ITGAV (116 kDa) and actin (42 kDa)


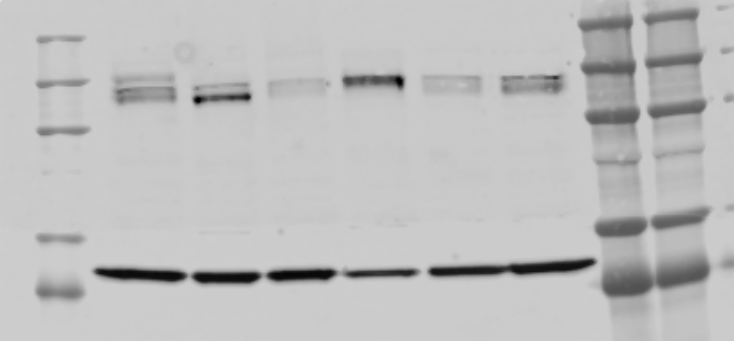


ITGA6 (120 kDa)


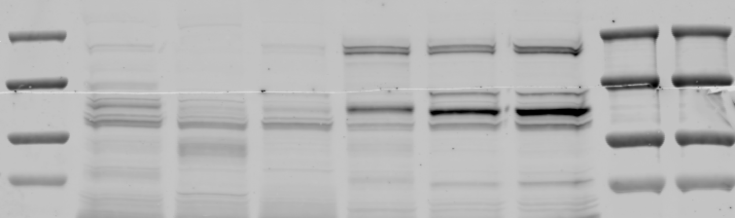


ITGA3 (120 kDa)


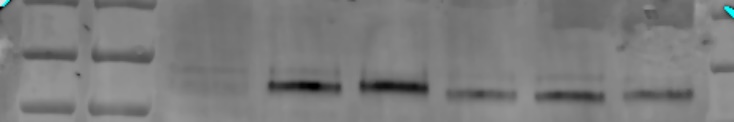


FAK (125 kDa)


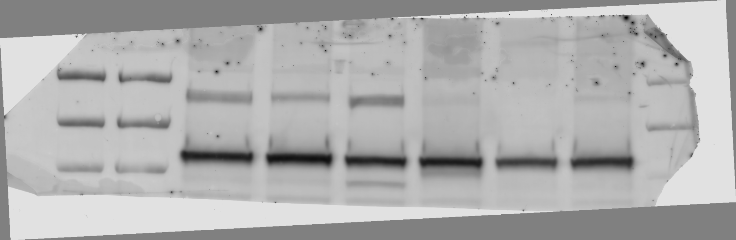


Membranes were cut at 50 kDa. They were first probed with ITGAV (116 kDa) and actin. The upper membrane was stripped and probed for ITGA6 (120kDa) next. The membrane was now cut at 150 kDa, stripped, and probed for ITGB4 (210 kDa) and ITGB3 (90 kDa). Finally, ITGA3 (120 kDa) and FAK (125 kDa) were probed on a separate membrane run in parallel.

The loading order from left to right: ladder, BT549, HS578T, MDAMB436, HCC1143, HCC1937, HCC1806, ladder, ladder, except tFAK and ITGA3 are mirror-images of this loading order.

Supplemental Figure 3.

ITGA3 (120 kDa) & Actin (42 kDa)


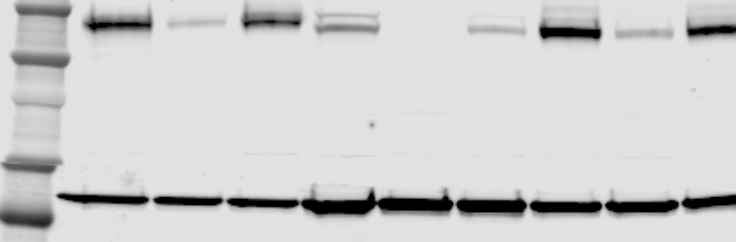


ITGA6 (120 kDa)


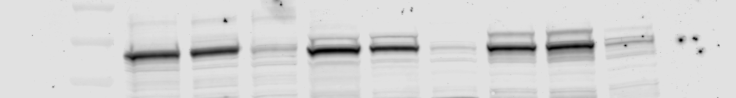


ITGA3 (120 kDa, red) & ITGA6 (120 kDa, green)


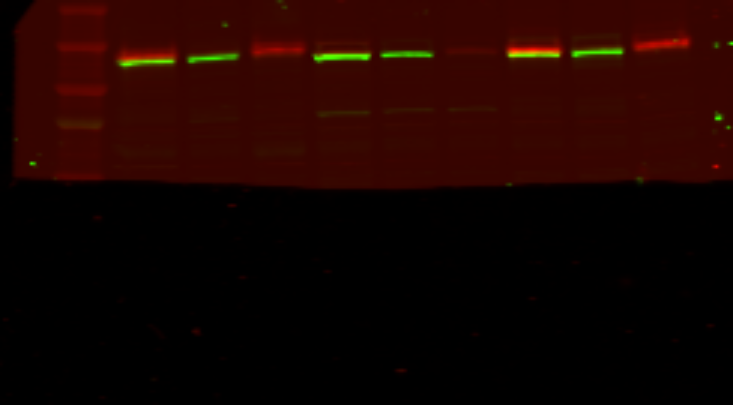


ITGB4 (210 kDa) & Actin (42 kDa)


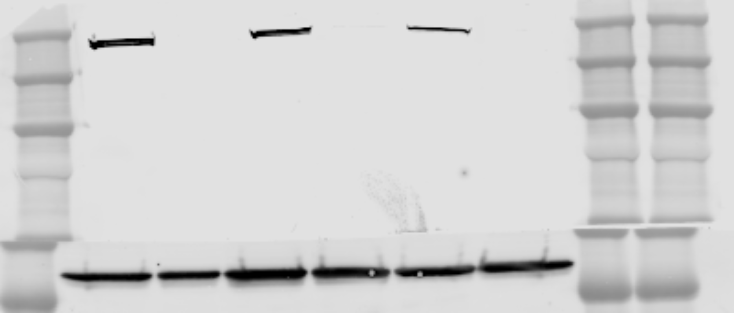


ITGA3 and ITGA6 were imaged together (ITGA3 and ITGA6 antibodies are made in mouse and rabbit respectively, enabling simultaneous exposure using fluorescent antibodies on the Li-COR Odyssey). To acquire actin, the membrane was cut above 50 kDa and the lower portion of the membrane was probed with the anti-actin antibody. The ITGB4 knockdown was a separate experiment with a separate blot. Again, the membrane was cut and the top portion was probed for ITGB4 and the bottom portion was probed for actin. Loading order of cell lines for the ITGA3 and ITGA6 blots is HCC1143, HCC1806, and HCC1937. Each cell line has three lanes loaded in the order: non-targeting control, siITGA3, and siITGA6. For the ITGB4 blot, cell lines are loaded in the order HCC1143, HCC1806, and HCC1937, with each cell line having two lanes loaded in the order: non-targeting control and siITGB4.

Supplemental Figure 5

ITGA3 (120 kDa)


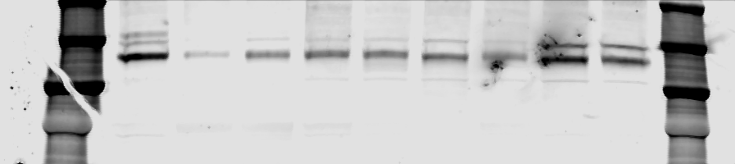


ITGA6 (116 kDa) and ITGB4 (210 kDa)


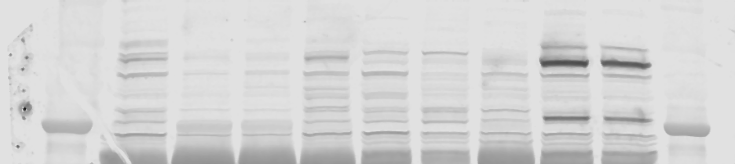


ITGB4 (210 kDa)


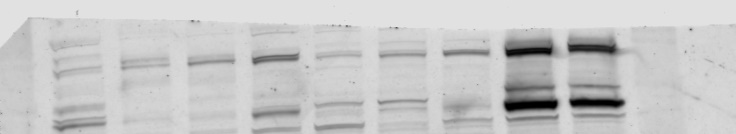


ITGB3 (90 kDa)


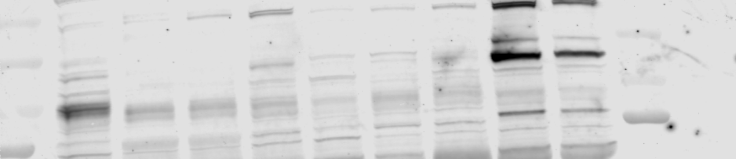


ITGAV (120 kDa)


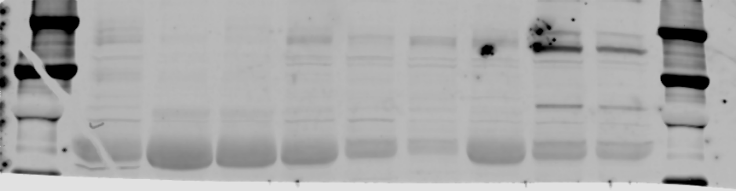


Actin


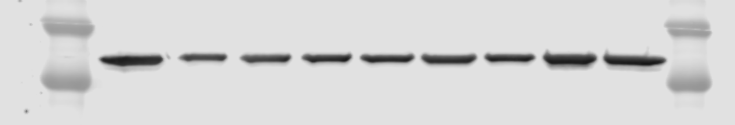


ITGA3, ITGA6, and ITGB4 were imaged together (ITGA3 and ITGA6 antibodies are made in mouse and rabbit respectively, enabling simultaneous exposure using fluorescent antibodies on the Li-COR Odyssey). To acquire actin, the membrane was cut above 50 kDa and the lower portion of the membrane was probed with the anti-actin antibody. The upper membrane was stripped and ITGB4 was reprobed. This membrane was again stripped and incubated with ITGB3. Finally, the membrane underwent an additional stripe-probe cycle to acquire ITGAV. Loading order: ladder, BT549, T47D, mCF7, ZR751, EFM19, HCC1428, MDAMB175VII, HCC1806, HCC1806, ladder.
