# Supplementary material for: Cilengitide sensitivity is predicted by overall integrin expression in breast cancer
Source: Breast Cancer Res. 2024 Dec 20;26:187. doi: 10.1186/s13058-024-01942-2 (PMC11660856; doi:10.1186/s13058-024-01942-2)
Supplement: Supplementary file 8 — Supplementary material 8. [file 13058_2024_1942_MOESM8_ESM.docx]

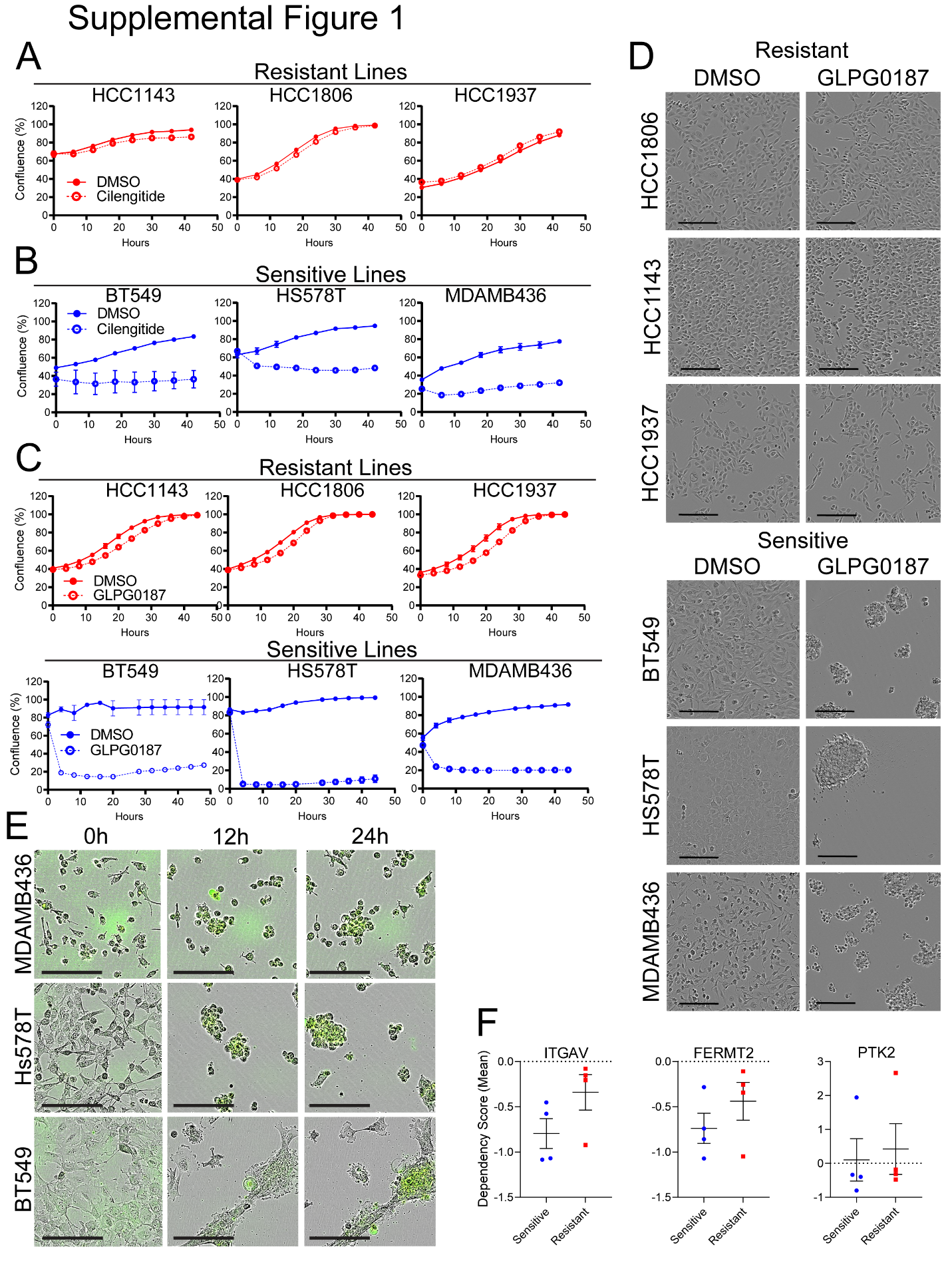


Supplemental Figure 1: A & B. Resistant (A) and sensitive (B) cell lines were plated in triplicate in 96 well plates, allowed to adhere overnight, and treated with DMSO (filled circles, solid line) or 5 µM cilengitide (open circles, dotted line) at time 0 h. Their confluence was monitored for 48 h at 4 h intervals. C. The same cell lines were cultured as in A & B, but were treated with DMSO (filled circles, solid line) or 2.5 µM GLPG0187 (open circles, dotted line) and cell growth was monitored at 4 h intervals for 48 h. D. Phase images of cilengitide-resistant (top) and -sensitve (bottom) cell lines 20 h after treatment with either DMSO (left) or GLPF0187 (right). Scale bar = 200 µM. E. Caspase 3/7 activation in sensitive cell lines 0, 12 and 24 h after dosing with 5 µM cilengitide was monitored using a dye that fluoresces when cleaved by activated caspase 3/7. Scale bar = 200 µm. F. The dependency values of the resistant and sensitive lines for a given gene were plotted together with the mean and SEM. No significant differences between the two groups were observed by t-test.


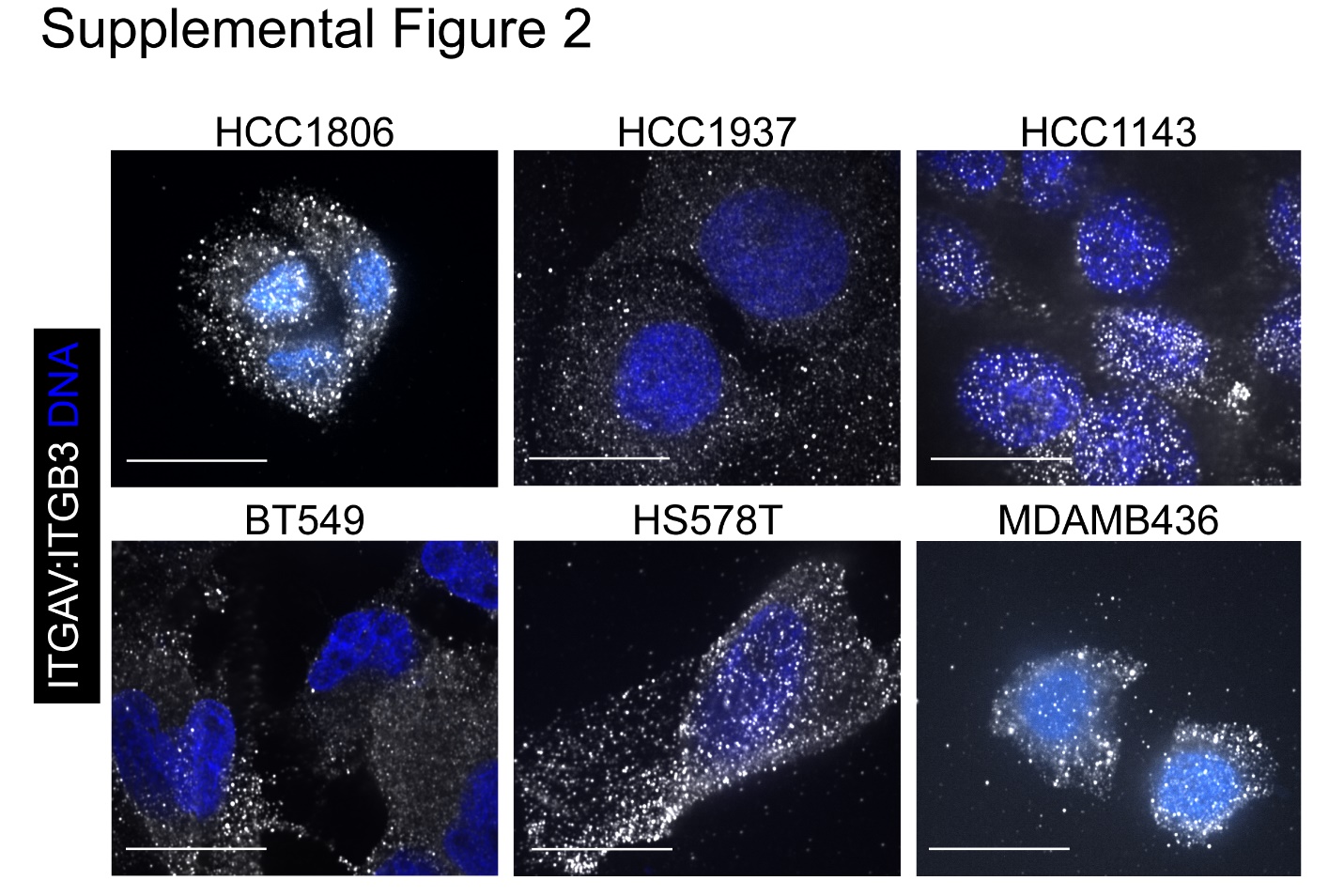


Supplemental Figure 2: The presence of dimer ITGAV:ITGB3 (white) was visualized in across cell lines. Cells were counterstained with DAPI (blue). Scale bar = 20 µm.


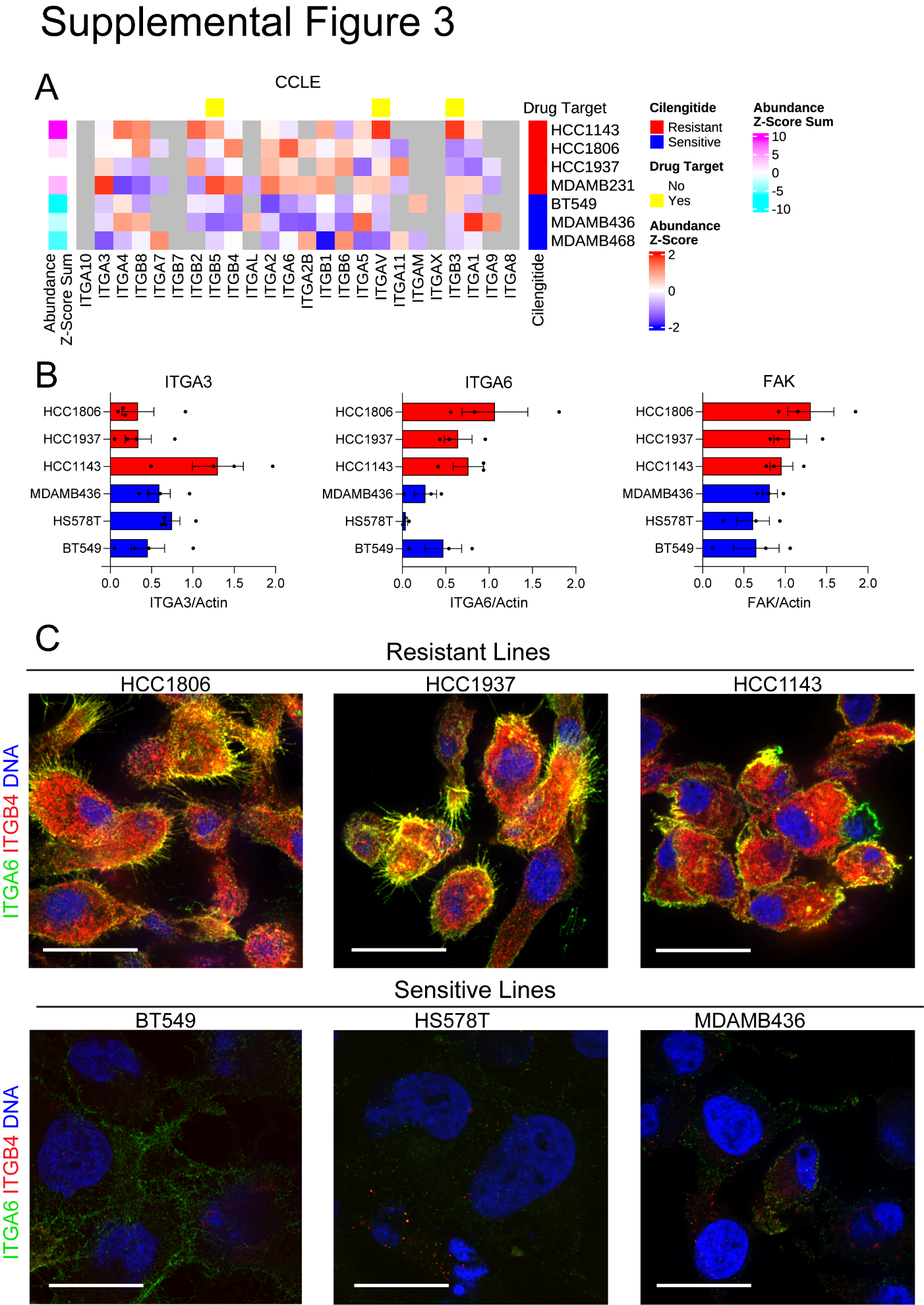


Supplemental Figure 3: A. A heatmap shows integrin abundance in cilengitide-resistant (red) and -sensitive (blue) cell lines from the CCLE proteomics dataset. Missing values are in gray, while higher values are in red and lower values in blue. The yellow boxes on top highlight cilengitide targets, while overall integrin abundance is shown in cyan and magenta to the left. B. Three independent cell protein lysates from each cell line were quantified for ITGA3, ITGA6, and total FAK (TFAK) and normalized to beta-actin. Resistant lines are colored in red, while sensitive lines are blue. The mean with S.E.M. is shown. C. ITGA6 (green) and ITGB4 (red) were visualized across cell lines. Cells were counterstained with DAPI (blue). Scale bar = 20 µm.


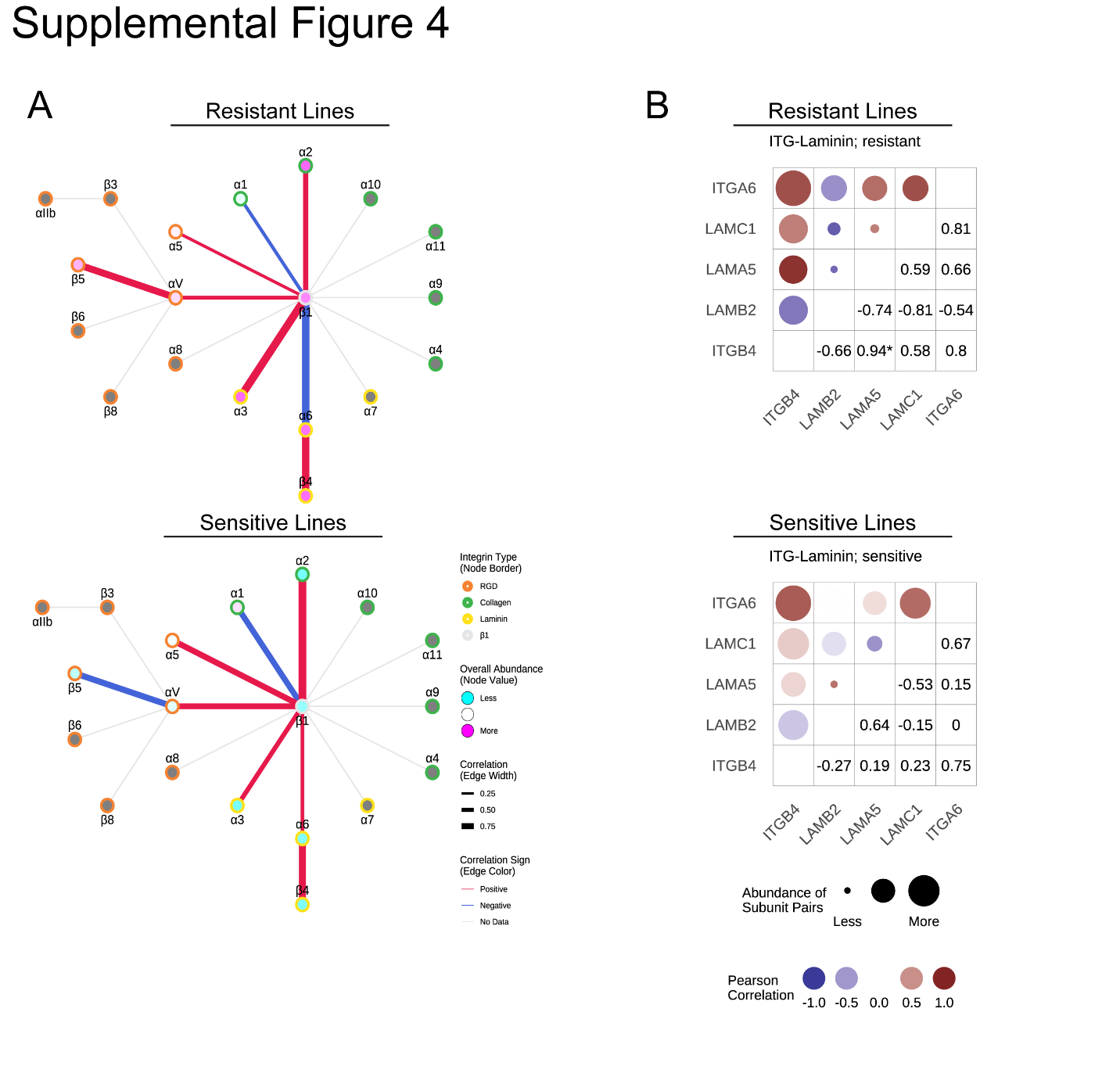


Supplemental Figure 4: A. A summary of integrin abundance (node value), correlation of expression of connected nodes (edge width, with red indicating a positive and blue a negative correlation), and ECM binding partners (integrin type) for resistant (left) and sensitive (right) cell lines. Connected integrins are known binding pairs. Gray nodes or lines indicate no data for that integrin or pairing, respectively. B. Correlation matrices of protein abundance from the BR80 dataset of the ITGA46:ITGB4:Laminin complex from the CORUM complex database in resistant (left) and sensitive (right) cell lines are presented. The circle colors denote positive (red) or negative (blue) Pearson values (shown), while the circle size indicates the relative abundance of the subunit pair.


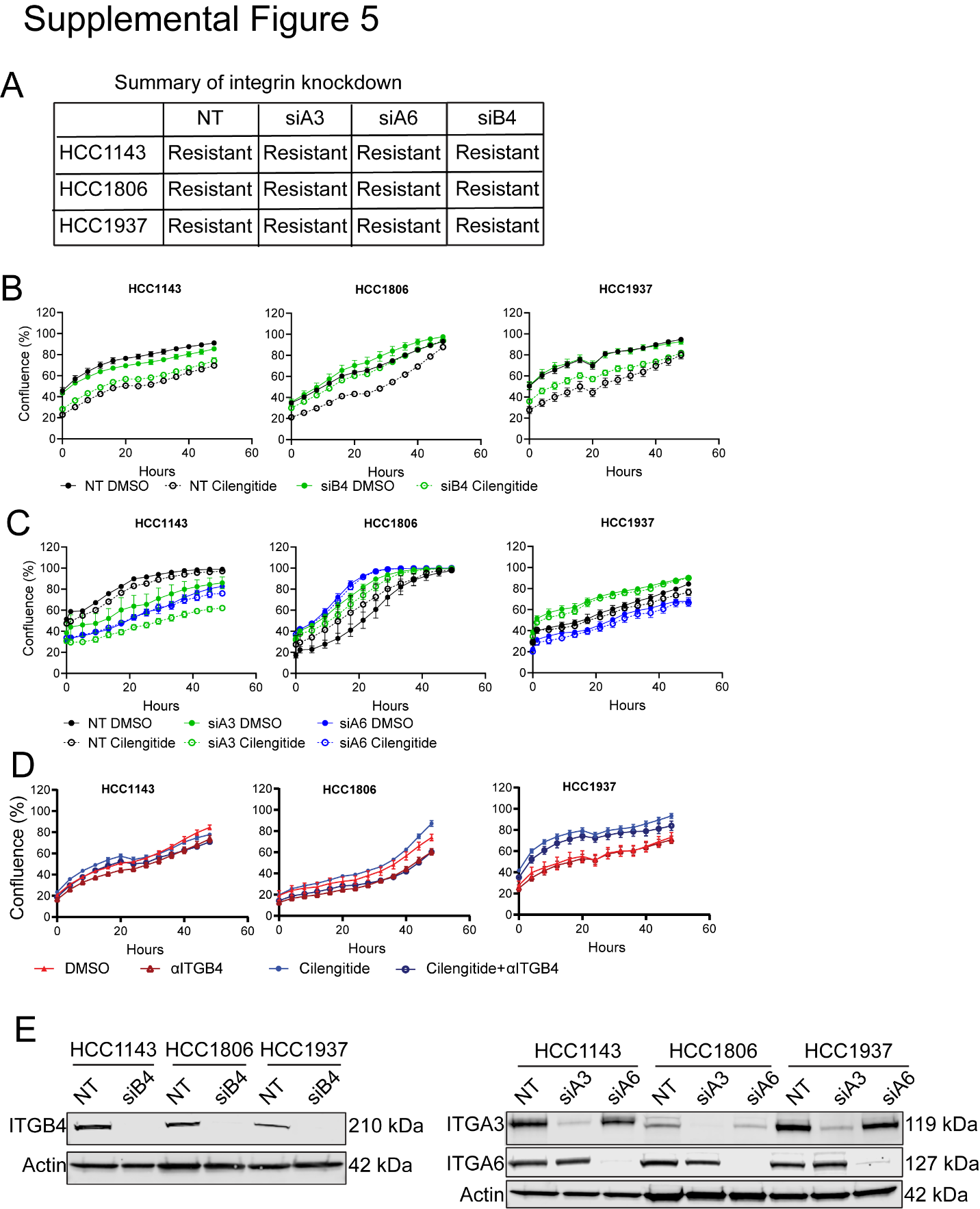


Supplemental Figure 5: A. A table summarizes siRNA knockdown experiment results using smartpool siRNAs B. Cells transfected with either non-targeting (NT, black) or ITGB4-targeting (siB4, green) smartpool siRNAs were plated in triplicate and treated with DMSO (close circle, solid line) or 5 µM cilengitide (open circle, dotted line) 48h after transfection. Cell confluence was measured at 4 h intervals over 48 h. One of 2 replicate experiments is shown. C. Cells transfected with either non-targeting, ITGA3-, or ITGA6-targeting (NT in black, siA3 in green, siA6 in blue, respectively) smartpool siRNAs were plated in triplicate and treated with DMSO (close circle, solid line) or 5 µM cilengitide (open circle, dotted line) 48h after transfection. Their confluence was monitored at 4 h intervals over 48 h. One of 2 replicate experiments is shown. D. HCC1143, HCC1806, and HCC1937 cells plated in triplicate were treated with 10 µg/ml of an ITGB4 blocking antibody (darker shades) in combination with DMSO (red triangles) or 5 uM cilengitide (blue circles) and their confluence was monitored at 4 h intervals over 48 h. E. Integrin knockdown was confirmed by immunoblotting. Lysates were made 48 h post transfection with smartpool siRNA against ITGB4 (siB4), or 3 days post transfection for ITGA3 or ITGA6 knockdown (siA3 or siA6, respectively). Knockdown conditions were compared to cells transfected with a non-targeting control siRNA (NT). Beta-actin was used as a loading control.


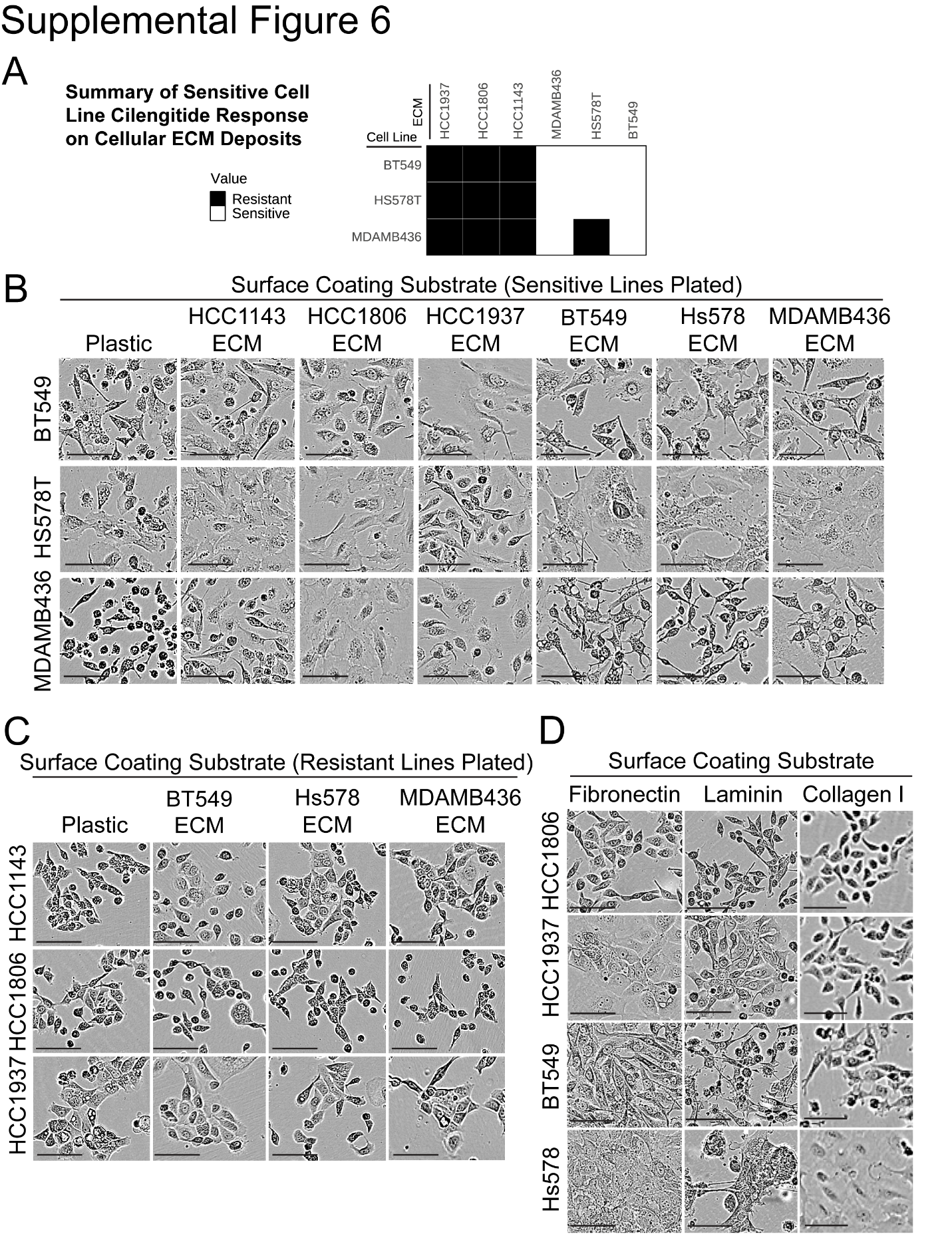


Supplemental Figure 6: A. A summary graphic shows the results of cell line ECM plating experiments in which cell lines were plated on ECM deposited by other cell lines. Black boxes indicate conditions in which cell lines were resistant to cilengitide. B-D. bright field images of cell lines plated on different substrates were acquired prior to treatment to examine changes in cell morphology: (B) sensitive lines plated on the ECM protein indicated above, (C) resistant lines plated on sensitive cell line ECM, and (D) sensitive (BT549, HS578T) and resistant (HCC1806, HCC1937) cell lines plated on fibronectin, laminin, or collagen. The morphology of cells plated on plastic is shown for comparison (B & C). Scale bars = 100 µm.


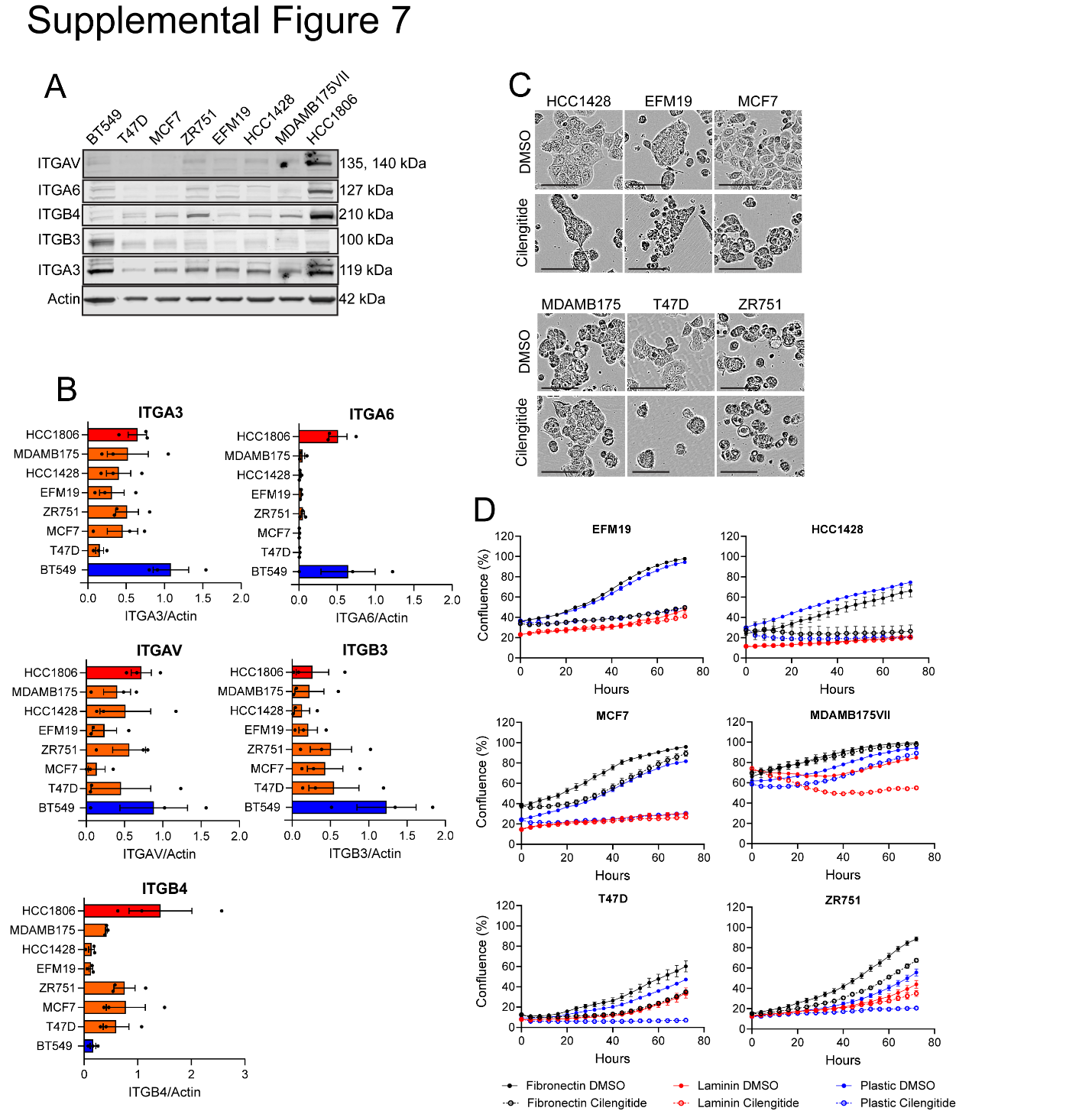


Supplemental Figure 7: A. Immunoblots show integrin expression in luminal cell lines, with beta-actin serving as a loading control. The sensitive and resistant lines BT549 and HCC1806 are included as references. B. Three independent cell protein lysates from each cell line were quantified for the ITGA3, ITGA6, ITGAV, ITGB3, and ITGB4, and normalized to beta-actin. The resistant line HCC1806 is colored in red, the sensitive line BT549 is colored in blue, and the ER+ lines are in orange. The mean with S.E.M. is shown. C. Bright field images of luminal breast cancer cell lines treated with DMSO or 5 µM cilengitide show cell rounding in the presence of cilengitide. D. Luminal cell lines were plated in triplicate on fibronectin (black), laminin (red), or plastic (blue), treated with DMSO (closed circles, solid lines) or 5 µM cilengitide (open circles, dashed lines) and their confluence was monitored at 4 h intervals for 72 h. In all cases, a representative of 3 replicate experiments is shown.
